# CmNDB1 and a Specific Domain of CmMYB1 Negatively Regulate CmMYB1-Dependent Transcription of Nitrate Assimilation Genes Under Nitrogen-Repleted Condition in a Unicellular Red Alga

**DOI:** 10.3389/fpls.2022.821947

**Published:** 2022-03-11

**Authors:** Baifeng Zhou, Hiroki Shima, Kazuhiko Igarashi, Kan Tanaka, Sousuke Imamura

**Affiliations:** ^1^School of Life Science and Technology, Tokyo Institute of Technology, Yokohama, Japan; ^2^Laboratory for Chemistry and Life Science, Institute of Innovative Research, Tokyo Institute of Technology, Yokohama, Japan; ^3^Department of Biochemistry, Tohoku University Graduate School of Medicine, Sendai, Japan; ^4^NTT Space Environment and Energy Laboratories, Nippon Telegraph and Telephone Corporation, Tokyo, Japan

**Keywords:** transcription factor, nitrogen, nitrogen-repleted, red alga, nitrate assimilation

## Abstract

Nitrogen assimilation is an essential process that controls plant growth and development. Plant cells adjust the transcription of nitrogen assimilation genes through transcription factors (TFs) to acclimatize to changing nitrogen levels in nature. However, the regulatory mechanisms of these TFs under nitrogen-repleted (+N) conditions in plant lineages remain largely unknown. Here, we identified a negative domain (ND) of CmMYB1, the nitrogen-depleted (−N)-activated TF, in a unicellular red alga *Cyanidioschyzon merolae*. The ND deletion changed the localization of CmMYB1 from the cytoplasm to the nucleus, enhanced the binding efficiency of CmMYB1 to promoters of nitrate assimilation genes, and increased the transcripts of nitrate assimilation genes under +N condition. A pull-down assay using an ND-overexpressing strain combined with liquid chromatography-tandem mass spectrometry (LC-MS/MS) analysis helped us to screen and identify an unknown-function protein, the CmNDB1. Yeast two-hybrid analysis demonstrated that CmNDB1 interacts with ND. Similar to ND deletion, *CmNDB1* deletion also led to the nucleus localization of CmMYB1, enhanced the promoter-binding ratio of CmMYB1 to the promoter regions of nitrate assimilation genes, and increased transcript levels of nitrate assimilation genes under +N condition. Thus, these presented results indicated that ND and CmNDB1 negatively regulate CmMYB1 functions under the +N condition in *C. merolae*.

## Introduction

Nitrogen is a component of most basic macromolecules, including proteins and nucleic acids. Thus, it is an essential element for all living organisms. Plants uptake nitrogen sources, mainly ammonium and nitrate through ammonium transporter (AMT) and nitrate transporter (NRT), respectively, from their growing environments. These nitrogen sources are then assimilated into amino acids through nitrogen assimilation enzymes, and these amino acids would be further utilized (Crawford and Forde, 2002).

Plants alter their gene expression to acclimate to the changing nitrogen level in the environment. Several studies have reported that transcription factors (TFs) positively regulate in controlling the expressions of nitrogen assimilation genes under nitrogen-deficient or −N conditions. In *Arabidopsis*, recent studies have uncovered that the Nodule INception (NIN)-Like Protein 6 and 7 (NLP6, NLP7), could positively regulate the transcripts of *AtNRT1.1*, as well as the gene-encoding nitrate reductase (NR), *AtNIA1* and *AtNIA2*, under −N conditions through forming a complex with TCP20 in the nucleus (Marchive et al., 2013; Guan et al., 2017). Although NLP6 and NLP7 have been originally identified as TFs that are involved in the regulation of primary nitrate response (Konishi and Yanagisawa, 2013; Marchive et al., 2013), the inactivation mechanism of −N-activated TFs under +N condition is not well studied. In rice, through the gene expression analysis, recent research has demonstrated that bZIP-type TFs, bZIP11 and bZIP41, positively regulate expression of *OsNRT2.3* and gene-encoding glutamine synthetase (GS), *OsGS1;1*, under nitrogen-deficient condition but not +N condition in roots (Ueda et al., 2020). The mechanism underlying the low transcription of bZIP11&41-mediated *OsNRT2.3* and *OsGS1;1* under +N condition remains unknown. Although functions of specific regions of the −N-activated TFs must be important for understanding the regulatory mechanism of these TFs under +N conditions, this point is unclear, so far. Furthermore, the regulators affecting the −N-activated TFs function under +N conditions in the plant kingdom are largely unknown.

*Cyanidioschyzon merolae* is a unicellular red alga, which was isolated from a hot spring in Italy (Kuroiwa, 1998). *C. merolae* has extremely simple cellular architectures and a minimally redundant small genome. *C. merolae* could be used as an excellent model of photosynthetic eukaryote for various research studies (Ohta et al., 1998, 2003; Matsuzaki et al., 2004; Nozaki et al., 2007). In our previous study, we have identified an R2R3-type MYB TF, CmMYB1, which is indispensable for −N-responsible transcription of nitrogen assimilation genes (Imamura et al., 2009). However, the mechanism underlying the regulation of CmMYB1 under the +N condition remains to be uncovered. Based on the advantage of *C. merolae* and the clear evidence of the −N-activated central player, CmMYB1, it is reasonable to use *C. merolae* for investigating the regulatory mechanism of CmMYB1.

In the present study, we revealed a critical region of CmMYB1, which negatively controls CmMYB1 functions under the +N condition. Furthermore, we identified and characterized an unknown-function protein, CMR469C (named CmNDB1 in this study), which negatively controls the function of CmMYB1 under the +N condition in *C. merolae*.

## Materials and Methods

### Strain and Growth Conditions

*C. merolae* T1, BZU1, BZ1, CmMYB1 partially truncated transformants (pPD1–310, pPD1–380, pPD1–460, pPD1–99, 201–523, pPD1–523, and pPD1–310, 381–523), MΔ95a and BZ6 strains were grown at 40°C under continuous white light (50 μmol m^–2^ s^–1^) in a liquid MA2 medium [containing 40 mM (NH_4_)_2_SO_4_; Imamura et al., 2010] at pH 2.5 bubbling with air supplemented with 2% CO_2_. For cultivation of T1, BZ1, and MΔ95a, about 0.5-mg ml^–1^ uracil and 0.8-mg ml^–1^ 5-fluoroorotic acid (5-FOA) were added to the MA2 medium.

### Construction of *CmMYB1* Knockout Strain, BZU1, and BZ1

Using the primer sets MYB1pro_F (listed in Supplementary Table 1), MYB1pro_R and MYB1ter_F, MYB1ter_R and KOD One Master Mix (Toyobo, Osaka, Japan), –1499 to +0 bp of *CmMYB1* promoter (+1 indicates the site of the start codon), and +1,570 to +3,070 bp of *CmMYB1* terminator were amplified with *C. merolae* genomic DNA as templates and purified by Wizard^®^ SV Gel and PCR Clean-up System (Promega, Madison, WI, United States). Purified *CmMYB1* promoter and terminator DNAs were then cloned into *Stu*I (Takara, Shiga, Japan)-digested pMKTf (Takemura et al., 2018) to create pMKT_CmMYB1_KO plasmid using the Gibson Assembly Master Mix (New England Biolabs, Ipswich, MA, United States). The DNA for *CmMYB1* knockout transformation was amplified using the primer set MYB1tf_F and MYB1tf_R and purified as mentioned above. The transformation using *C. merolae* T1 strain (Taki et al., 2015) was performed as described previously (Takemura et al., 2018). To get the uracil auxotrophy strain of BZU1, BZ1, approximately 1 × 10^8^ BZU1 strain cells were mixed with 1-ml 50% m/v cornstarch solution and spread on an MA2 Gellan Gum plate containing 0.5-mg ml^–1^ uracil and 0.8 mg ml^–1^ 5-FOA. The plate was incubated in a bag with Aneropack CO_2_ (Mitsubishi Gas Chemical Co., Inc., Tokyo, Japan) for 4 weeks, and colonies were picked up into a 2-ml MA2 medium supplemented with 0.5-mg ml^–1^ uracil and 0.8-mg ml^–1^ 5-FOA for 2 weeks further. The PCR analysis was performed to select the desired transformants using the primers listed in Supplementary Table 1.

### RNA Preparation and Northern Blot Analysis

Total RNA preparation and Northern blot analysis were performed as described previously (Imamura et al., 2008). Gene-specific probes for Northern blot analyses were generated with specific primers (listed in Supplementary Table 2) and the *C. merolae*-genomic DNA as templates.

### Construction of CmMYB1 Amino Acids Truncated Strains

Plasmids expressing truncated types of CmMYB1 were constructed using primers listed in Supplementary Table 1. −1,499 to +0 bp of the *CmMYB1* promoter together with *CmMYB1* ORF and +1,570 to +3,070 bp of the *CmMYB1* terminator were first amplified using the primer sets MYB1pro_F2 (listed in Supplementary Table 1), MYB1orf_R, MYB1ter_F, and MYB1ter_R, respectively, with KOD One Master Mix (Toyobo, Osaka, Japan) with *C. merolae*-genomic DNA as templates. Then, they were purified by Wizard^®^ SV Gel and PCR Clean-up System (Promega, Madison, WI, United States). Two fragments: the *CmMYB1* promoter together with ORF and the *CmMYB1* terminator were cloned into separate two *Stu*I sites of pMKTf (Takemura et al., 2018) to construct pMKT_CmMYB1 plasmid. Plasmids—pMKT_1-310 (TEV_F, 1_310_R), pMKT_1-380 (TEV_F, 1_380_R), pMKT_1-460 (TEV_F, 1_460_R), pMKT_1-99, 201-523 (MYB1db_F, MYB1db_R), pMKT_1-310, 381-523 (MYB1ne_F, MYB1ne_R)—were constructed using the pMKT_CmMYB1 plasmid as templates and primer sets indicated in the individual brackets above. The BZ1 strain was cultured under normal culture conditions supplemented with 0.5-mg ml^–1^ uracil and 0.8-mg ml^–1^ 5-FOA to reach OD_750_ above 3. Then, BZ1 cells were diluted into a 50-ml MA2 medium supplemented with 0.5-mg ml^–1^ uracil and 0.8-mg ml^–1^ 5-FOA to give an OD_750_ to 0.4 and cultured in the same culture condition as mentioned above until OD_750_ reached 0.5 to 0.7. BZ1 cells were centrifuged and resuspended in a 180-μl MA2 medium containing 0.5-mg ml^–1^ uracil in a 1.5-ml microtube. The transformation using *C. merolae* BZ1 strain was performed as described previously (Takemura et al., 2018).

### Protein Extraction and Immunoblot Analysis

*C. merolae* logarithmic growth cells (25 ml at OD_750_ = 0.5) were collected, and a 250-μl lysis buffer [50-mM Tris-HCl, pH 7.5, 150-mM NaCl, 10% v/v Glycerol, a complete protease inhibitor EDTA-free cocktail (Roche, Basel, Switzerland), a phosphatase inhibitor cocktail (Nacalai Tesque, Inc., Kyoto, Japan)] was added into the cell pellet followed by 0.15-g glass beads (∅ ≤ 106 μm, Sigma-Aldrich, MA, United States). Cells were disrupted by vortexing (scale 10, 5 min, TurboMix attached to Vortex Genie 2, Scientific Industries, New York, NY, United States), and supernatant (hereafter, a total soluble protein fraction) was collected by centrifuging at 15,000 rpm for 20 min at 4°C and stored at −80°C until use. Protein samples were boiled at 95°C for 5 min and later cooled on ice for 2 min. The proteins were separated by 10% SDS-PAGE and transferred to a polyvinylidene fluoride (PVDF, Millipore) membrane (0.45-μm pore size, Millipore, Burlington, MA, United States). The membrane was blocked with 5% w/v skim milk in 0.1% TBS-Tween at 4°C overnight. In case of detecting FLAG-fused CmMYB1, the membrane was briefly washed three times with 0.1% TBS-Tween before being incubated with an anti-FLAG antibody (Wako, Osaka, Japan, dilution rate: 1/5,000) for 1 h at room temperature. Next, the membrane was washed as mentioned above and incubated with antimouse IgG (H + L) and an HRP conjugate antibody (Promega, Madison, WI, United States, dilution rate: 1/5,000) for 1 h at room temperature. The washing step was repeated before visualization using the ImmunoStar Zeta (Fujifilm, Tokyo, Japan).

### Dot Blot Analysis

A total soluble protein fraction was collected as described in section “Protein extraction and immunoblot analysis.” PVDF membrane was firstly treated with methanol for 60 s and washed with RO water two times. Then, a blotting buffer (25-mM Tris base, 192-mM glycine, 20% v/v methanol) was used for PVDF membrane incubation for 5 min; a 16.6-μg/sample total soluble protein fraction was used. The membrane was dried for 10 min and incubated in a blocking solution (5% w/v skim milk in 0.1% TBS-Tween) at 4°C overnight. The remaining steps were the same as mentioned in *immunoblot analysis*.

### cDNA Synthesis and Quantitative Real-Time PCR Analysis

Total RNA was prepared as described previously (Imamura et al., 2008). Genome DNA was removed, and cDNA was synthesized by ReverTra Ace^®^ qPCR RT Master Mix with gDNA Remover (Toyobo, Osaka, Japan) using 500 ng RNA of each sample as input. Synthesized cDNA was then subjected to Quantitative Real-Time PCR (qRT-PCR) analysis. qRT-PCR analysis was performed as described previously (Imamura et al., 2008). Primers used for qRT-PCR analyses are listed in Supplementary Table 2.

### Immunostaining Analysis

*C. merolae* logarithmic growth cells (10 ml at OD_750_ = 0.5) were collected and fixed with a cold fixation buffer (Ohnuma et al., 2008) and incubated at −30°C for 5 min. Fixed cells were then collected and resuspended in methanol and stored at −30°C until use. The cells were washed with 1 × PBS and blocked with 5% v/v, blocking one solution (hereafter, blocking solution, Nacalai Tesque, Inc., Kyoto, Japan) for 30 min at room temperature. The cells were washed with blocking solution and incubated with an anti-FLAG antibody (Wako, Osaka, Japan, a dilution rate: 1/100) in blocking solution at 4°C overnight. The cells were washed with blocking solution and incubated with an Alexa Fluor 488 donkey antimouse antibody (Thermo Fisher Scientific, Waltham, MA, United States, a dilution rate: 1/100) in blocking solution at room temperature for 1 h while avoiding light. The cells were then washed with blocking solution, and the fluorescence signal was captured by fluorescence microscopy (BX-51, Olympus, Tokyo, Japan). Data were treated by Adobe Photoshop 2021 to enhance the contrast and light intensity of the fluorescence from the sample cells.

### Chromatin Immunoprecipitation Analysis

*C. merolae* logarithmic growth cells (25 ml at OD_750_ = 0.5) were collected and fixed as described previously (Imamura et al., 2008). The fixed cells were resuspended in a 1-ml ChIP lysis buffer [50-mM Tris-HCl, 1-mM EDTA, pH 8.0, 140-mM NaCl, 0.1% SDS, 1% Triton X-100, 0.1% sodium deoxycholate, Complete Mini, EDTA-free, a protease inhibitor (Roche, Basel, Switzerland)] and disrupted by sonication (Sonifier 250 advanced, Branson, Emerson, Tokyo, Japan) with output 3, 50% duty for 18 cycles. The supernatant (chromatin solution) was collected by centrifugation and stored at −80°C until use. About 175-μl Slurry protein G Sepharose 4 Fast Flow (Roche, Basel, Switzerland) with a 900-μl ChIP lysis buffer was added to 100-μl chromatin solution, and the samples were rotated at 4°C overnight. The supernatant was collected by removing protein G Sepharose 4 Fast Flow using Poly-Prep Chromatography Columns (Bio-Rad, Hercules, CA, United States) and incubated with a 2.5-μg/sample anti-FLAG antibody (Wako, Osaka, Japan) at 4°C overnight. The samples were further incubated for 5 h at 4°C after adding 30-μl/sample Dynabeads protein G (Thermo Fisher Scientific, Waltham, MA, United States). Dynabeads were collected by a magnetic stand and sequentially washed as described previously (Imamura et al., 2008). Together with 20-μg/sample RNase A (Nippon Gene, Tokyo, Japan), a 500-μl/sample ChIP direct elution buffer (Imamura et al., 2008) was added to Dynabeads, and the samples were incubated at 65°C overnight. The samples were further incubated at 37°C for 1 h after adding 40-μg/sample proteinase K (Thermo Fisher Scientific, Waltham, MA, United States). The supernatant was collected, and DNA was extracted using phenol:chloroform:isoamyl alcohol (25:24:1) followed by ethanol precipitation. Finally, the pellet was dissolved in 25-μl Milli-Q™ (Millipore, Burlington, MA, United States) water and used for qRT-PCR analyses. Primers used for qRT-PCR analyses are listed in Supplementary Table 2.

### Construction of Negative Domain-Overexpressing Strain and Control Strain (TFc)

The pSUGA_CmMYB1_1-380 was first created with primer set MYB1OE380_F (listed in Supplementary Table 1), MYB1OE380_R with pO250-CmMYB1 (Zhou et al., 2021) as a template using the Gibson Assembly Master Mix (New England Biolabs, Ipswich, MA, United States). Then, primer sets MYB1TFc_F, MYB1TFc_R, and MYB1ND_OE_F, MYB1ND_OE_R were used to create pSUGA_CmMYB1_1−9, pSUGA_CmMYB1_1−9, and 311−380 with pSUGA_CmMYB1_1−380 as templates, respectively. The T1 strain was transformed with pSUGA_CmMYB1_1−9 and pSUGA_MYB1_1−9, 311−380, as described previously (Takemura et al., 2018), and transformants were spread and grown on an MA2 gellan gum plate. The PCR using in the primer set listed in Supplementary Table 1 was performed to get the correct transformants. Each cloned protein was fused with a FLAG-tag, and the expression was regulated by the strong *APCC* promoter (Fujii et al., 2013).

### Pull-Down Assay and Liquid Chromatography-Tandem Mass Spectrometry Analysis

Proteins from Negative Domain-Overexpressing Strain (ND_OE) and TFc were extracted as described in section “Protein extraction and immunoblot analysis.” From each sample, the 750-μg protein was subjected to the pull-down assay with 50-μl/sample anti-FLAG antibody magnetic beads (Wako, Osaka, Japan). The beads were washed six times with a lysis buffer (see section “Protein extraction and immunoblot analysis”) and were boiled for 5 min in an SDS sample buffer. The eluted proteins were separated by SDS-PAGE using a 5–20% polyacrylamide gradient gel (Oriental Instruments, Kanagawa, Japan) and were visualized by Coomassie brilliant blue staining. Each lane in the gel was entirely cut out, was transferred to a 14-ml round-bottom tube, and was shaken sequentially in 30, 50, and 100% acetonitrile solutions for destaining and dehydration. The gel strips were incubated in 25-mM ammonium bicarbonate containing 10-mM dithiothreitol (DTT) at 56°C for 1 h and then in 25-mM ammonium bicarbonate containing 55-mM acrylamide at room temperature for 1 h for reduction and alkylation of the sulfhydryl groups. The samples were washed in water and were then dehydrated in the acetonitrile solutions before tryptic digestion. The dried gel strips were reconstituted by a solution containing 90-ng Trypsin Gold (Promega, Madison, WI, United States), 50-mM ammonium bicarbonate, and 10% acetonitrile, and were incubated overnight at 37°C. The resulting tryptic peptides were eluted three times in 75% acetonitrile and 1% formic acid. After concentration in a speedvac, the samples were finally dissolved in 45 μl of 0.5% formic acid. From each sample, 15 μl was subjected to liquid chromatography-tandem mass spectrometry (LC-MS/MS) using an Orbitrap Fusion mass spectrometer equipped with an Easy-nLC 1,000 high-performance liquid chromatography (HPLC) system (Thermo Fisher Scientific, Waltham, MA, United States). The peptides were separated on a C18 tip column (a 75-μm inner diameter and 10-cm length, Nikkyo Technos, Tokyo, Japan) at a 300-ml min^––1^ flow rate with a linear gradient generated by aqueous solvent A (0.1% formic acid in water) and organic solvent B (0.1% formic acid in acetonitrile): 5% B to 35% B in 40 min, to 45% B in 46 min, to 95% B in 48 min, 95% B from 48 to 49 min, and, finally, to 5% B in 50 min. MS^1^ scans from *m*/*z* = 321--1,500 were performed in the Orbitrap mass spectrometer with the resolution set to 120,000 with lock masses at *m*/*z* = 445.12003 and 391.28429, which was followed by the acquisition of higher energy collisional dissociation (HCD)-MS^2^ using the ion trap. The settings for the MS^2^ scans were as follows: an intensity threshold = 1,000, charge states = + 2 to + 6, isolation width = 1.2 *m*/*z*, AGC target = 5,000, maximum ion injection time = 50 ms, normalized collision energy = 35%, and dynamic exclusion enabled with 50-s exclusion duration. The MS/MS cycle time was set to 4 s. The MS/MS data were acquired over 55 min after the LC gradient was started. Peptide identification was done by searching the *C*. *merolae* protein database^1^ and a common contaminating protein list using a MASCOT search engine (version 2.6.0, Matrix Science, Mount Prospect, IL, United States). A maximum of four trypsin miscleavages were allowed. The peptide mass tolerance and MS/MS tolerance were set at 5 ppm and 0.5 Da, respectively. Propionamidated cysteine (+71.0371) was set as a fixed modification. Protein N-terminal acetylation (+42.0106 Da) and oxidation of methionine (+15.9949 Da) were considered as variable modifications. The Mascot ion score threshold was set to 0.05. Obtained MS/MS data are presented in Supplementary Table 3.

### Yeast Two-Hybrid Analysis

The open-reading frames (ORFs) of the top 15 candidates’ encoded genes picked up from LC-MS/MS analysis were amplified with the primer sets listed in Supplementary Table 4 [For Y2H activation domain (AD) vector construction] and *C. merolae* genomic DNA as templates. The PCR-amplified genes were then cloned into *Sma*I-digested pGADT7, respectively, using the Gibson Assembly Master Mix (New England Biolabs, Ipswich, MA, United States) to create each AD vector. To create the BD plasmid, the *CmMYB1* ORF was firstly amplified with the primer set MYB1y2h_F, MYB1y2h_R listed in Supplementary Table 4 [For Y2H-binding domain (BD) vector construction] and *C. merolae* genomic DNA as a template. The PCR-amplified *CmMYB1* ORF was then cloned into *Sma*I-digested pGBT9 using the same method mentioned above to create the pGBT9_CmMYB1 vector. The pGBT9_CmMYB1_1–380 plasmid was then created using the same method mentioned above with primer set MYB1y2h_381_F, MYB1y2h_381_R listed in Supplementary Table 4, and pGBT9_CmMYB1 was used as a template. Finally, BD plasmid pGBT9_ND was created using primer set MYB1ND_OE_F, MYB1ND_OE_R listed in Table S4, and pGBT9_CmMYB1_1–380 as a template. All the vectors (15 AD vectors and 1 BD vector) were transformed to yeast gold competent cells (Takara, Shiga, Japan) using Frozen-EZ Yeast Transformation II Kit (Zymo Research, Irvine, CA, United States) according to the manufacturer’s instructions. Transformants were incubated at 30°C for 1 week, and transformants containing AD vectors were mated with transformants containing BD vector, respectively, and spread on SD−LW [synthetic dextrose (SD), leucine (L), and tryptophan (W) lacking] plates. Resulted transformants were further diluted and spotted on SD−LWH (L, W, and histidine (H) lacking) with 5-Bromo-4-chloro-3-indolyl-α-D-galactopyranoside [(X-α-Gal) (Wako, Osaka, Japan) and Aureobasidin A (AbA) (Takara, Shiga, Japan) being added] and SD−LWHA (L, W, H, and alanine (A) lacking with X-α-Gal and AbA being added) plates and SD−LW plates and incubated at 30°C for 1 week. Photos of SD−LW plates were taken on the second incubation day. Photos of SD−LWH and SD−LWHA plates were taken on the 7th incubation day.

### Construction of FLAG-Fused CmMYB1 Strain, WM1

The DNA fragment used for transformation was constructed through three steps. Firstly, the primer set CAT_MCS_F (listed in Supplementary Table 1), NOS_ter_R was used to amplify Fragment_1 using KOD Plus NEO (Toyobo, Osaka, Japan), and pMKTf (Takemura et al., 2018) plasmid as a template. Secondly, primer set NOS_ter_CAT_F, URA_CAT_R was used to amplify Fragment_2 using KOD Plus NEO (Toyobo, Osaka, Japan) and pD-184 CAT plasmid (given by Dr. Fujiwara) as a template. Thirdly, primer sets MCS_J282C_orf_F, MCS_J282C_orf_R, and MCS_J282C_ter_F, MCS_J282C_ter_R were used to amplify CmMYB1_ORF DNA and CmMYB1_ter DNA, respectively, using KOD Plus NEO (Toyobo, Osaka, Japan) and *C. merolae* genomic DNA as templates, respectively. Then, Fragment_1 and Fragment_2 were used to create pMKT_CAT plasmid using the Gibson Assembly Master Mix (New England Biolabs, Ipswich, MA, United States). Restriction enzyme *Hpa*I was used to cut the pMKT_CAT plasmid, and two linear DNA fragments, “pMKT” DNA fragment and “CAT” DNA fragment, were obtained. Finally, “pMKT” DNA, “CAT” DNA, CmMYB1_ORF DNA, and CmMYB1_ter DNA were used to create pMKT_CAT_CmMYB1 plasmid using the Gibson Assembly Master Mix (New England Biolabs, Ipswich, MA, United States). The primer set MCS_J282C_orf_F, MCS_J282C_ter_R was used to amplify CmMYB1_CAT DNA using KOD Plus NEO (Toyobo, Osaka, Japan). The CmMYB1_CAT DNA was then transformed into a WT strain as described previously (Takemura et al., 2018), and transformants were cultured in an MA2 medium for 1 week and spread on an MA2 gellan gum plate containing 300 μg ml^–1^ chloramphenicol (Fujiwara et al., 2017). The PCR using primer sets listed in Supplementary Table 1 was performed to get correct transformants.

### Construction of Uracil Auxotrophy Strain of WM1, MΔ95a

The DNA fragment used for transformation was constructed through four steps. Firstly, the primer set URA5.3pro_KS_F (listed in Supplementary Table 1), URA5.3ter_SK_R was used to amplify Fragment_1, using KOD Plus NEO (Toyobo, Osaka, Japan) and *C. merolae* genomic DNA as a template. Secondly, Fragment_1 was inserted into the *Sma*I-digested pBluescript SK plasmid to create the pBluescript _URA5.3 plasmid. Thirdly, inverse PCR was performed using primer set URA5.3ORFdel_F, URA5.3ORFdel_R with pBluescript _URA5.3 plasmid as a template, and the amplified DNA fragment was directly used for creating the pBluescript _URA5.3KO plasmid with the Gibson Assembly Master Mix (New England Biolabs, Ipswich, MA, United States). Finally, the DNA fragment used for transformation was amplified using the primer set URA5.3pro_F, URA5.3ter_R with pBluescript _URA5.3KO plasmid as a template. The amplified DNA fragment was then transformed into the WM1 strain as described previously (Takemura et al., 2018), and transformants were spread and grown on an MA2 gellan gum plate supplied with 0.5-mg ml^–1^ uracil and 0.8-mg ml^–1^ 5-FOA. Sequencing analysis was performed using URA5.3_seq_F primer listed in Supplementary Table 1 to get the correct transformants.

### Construction of *CmNDB1* Knockout Strain, BZ6

The DNA fragment used for transformation was constructed through three steps. Firstly, the primer sets pCmNDB1pro_F (listed in Supplementary Table 1), pCmNDB1pro_R, and pCmNDB1ter_F, pCmNDB1ter_R were used to amplify Fragment_1 and Fragment_2, using KOD Plus NEO (Toyobo, Osaka, Japan) and *C. merolae* genomic DNA as templates, respectively. Secondly, Fragment_1 and Fragment_2 were inserted into the *Stu*I-digested pMKTm (Takemura et al., 2018) plasmid to create the pMKT_CmNDB1_KO plasmid. Thirdly, the DNA fragment used for transformation was amplified using the primer set CmNDB1_879pro_F, CmNDB1_651ter_R with pMKT_CmNDB1_KO plasmid as a template. The amplified DNA fragment was then transformed into the MΔ95a strain as described previously (Takemura et al., 2018), and transformants were spread and grown on an MA2 gellan gum plate. The PCR using primer sets listed in Supplementary Table 1 was performed to get the correct transformants.

### Construction of Myc-Fused CmNDB1 Strain, MR66

The DNA fragment used for transformation was constructed through three steps. Firstly, the primer sets CmNDB1orf_F (listed in Table S1), CmNDB1orf_R, and CmNDB1ter_F, CmNDB1ter_R were used to amplify Fragment_1 and Fragment_2, using KOD Plus NEO (Toyobo, Osaka, Japan) and *C. merolae* genomic DNA as templates, respectively. Secondly, Fragment_1 and Fragment_2 were inserted into the *Stu*I-digested pMKTm (Takemura et al., 2018) plasmid to create the pMKT_NDB1myc plasmid. Thirdly, the DNA fragment used for transformation was amplified using the primer set CmNDB1tf_F, CmNDB1tf_R with pMKT_NDB1myc plasmid as a template. The amplified DNA fragment was then transformed into the MΔ95a strain as described previously (Takemura et al., 2018), and transformants were spread and grown on an MA2 gellan gum plate. The PCR using primer sets listed in Supplementary Table 1 was performed to get the correct transformants.

## Results

### Identification of the Negative Element of CmMYB1 for Its Function

**FIGURE 1:**
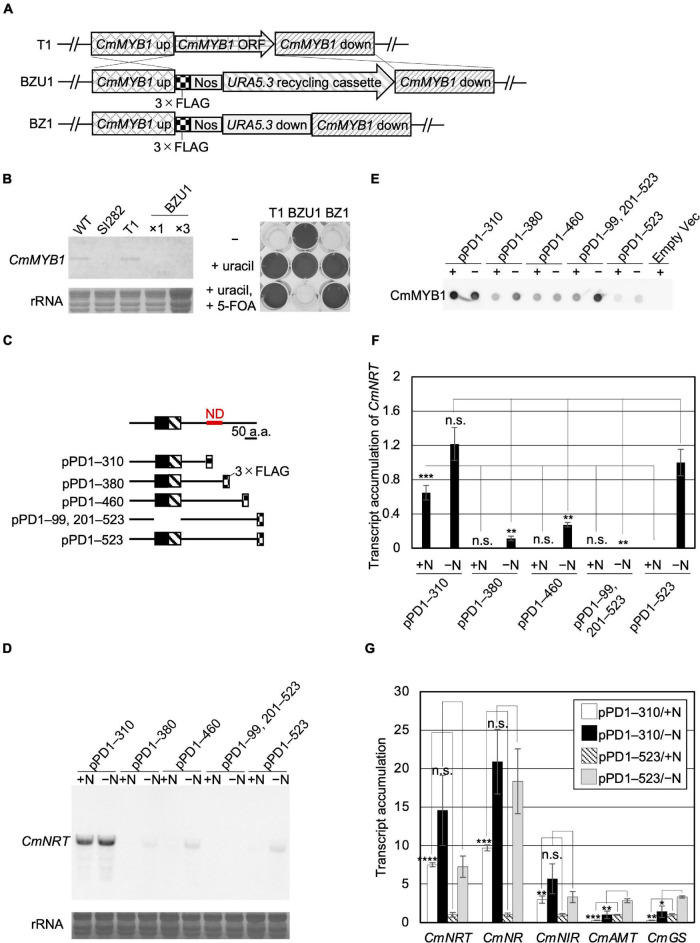
Acquisition of CmMYB1 partially truncated transformants and expression of nitrogen assimilation genes under nitrogen-repleted (+N)/nitrogen-depleted (−N) conditions. **(A)** Schematic representation of the *CmMYB1* ORF locus in the T1 (top panel), BZU1 (middle panel), and BZ1 (bottom panel) strains. “*CmMYB1* up” and “*CmMYB1* down” indicate the 1,500-bp upstream and downstream regions of *CmMYB1*, respectively, used for homologous recombination. “*CmMYB1* ORF” indicates the 1,569-bp open-reading frame of *CmMYB1*. “Nos” indicates the nopaline synthase (NOS) terminator. “Uracil recycling cassette” indicates a 3,234-bp fragment used for uracil auxotrophic transformant selection. “*URA5.3* down” indicates a 483-bp downstream region of *URA5.3*. **(B)** Confirmation of the transcript level of *CmMYB1* in BZU1 strain (left panel) and uracil auxotrophy of BZ1 strain (right panel). Left panel, BZU1, and T1 cells were harvested under normal culture condition, and total RNAs were prepared from the cells. Total RNAs (2.8 μg, ×1) were then subjected to Northern blot analysis with a specific probe for *CmMYB1*. Total RNAs (8.4 μg) from BZU1 cells were used as an overloaded sample (×3) in the analysis. rRNA-stained with methylene blue is shown as the loading control (bottom panel). SI282 strain is a *CmMYB1*-null strain whose host strain is M4, a uracil auxotrophy strain. T1, which was generated from WT, contains no *URA5.3* ORF. The right panel, stationary phase T1, BZU1, and BZ1 cells were diluted into OD_750_ = 0.1 and incubated for 7 days prior to photography. “-” represents an MA2 medium. “+uracil” represents an MA2 medium containing 0.5-mg ml^–1^ uracil. “+uracil, +5-FOA” represents an MA2 medium containing 0.5-mg ml^–1^ uracil and 0.8-mg ml^–1^ 5-fluoroorotic acid (5-FOA). **(C)** Schematic representation of the CmMYB1 partially truncated transformants. Black and hatched boxes indicate the R2- and R3-type MYB domain, respectively. As an example, pPD1–310 represents the transformant, which expresses +1 to +310 amino acids (+1 indicates the start position of translation) of CmMYB1 with three-time repeats of the FLAG epitope tag (3 × FLAG). ND is short for “negative domain.” **(D)** Transcript levels of *CmNRT* under the +N/−N conditions in CmMYB1 partially truncated transformants. Cells were treated under the +N/−N conditions for 4 h, and total RNAs were prepared from the cells. Total RNAs (4.5 μg) were used in Northern blot analysis with a specific probe for *CmNRT*. Same experiments were performed three times, independently. **(E)** Protein levels of FLAG-fused CmMYB1 under the +N/−N conditions in CmMYB1 partially truncated transformants. Aliquots containing 16.6 μg of total proteins harvested from the *C. merolae* cells under the same condition as in **(D)** were dropped on a hydrophilized PVDF membrane and analyzed with an anti-FLAG antibody (Wako, Osaka, Japan). Proteins extracted from empty vector transformants (Empty Vec) were used as a negative control. Same experiments were performed three times, independently. **(F)** Transcript accumulation of *CmNRT* in CmMYB1 partially truncated transformants. ImageJ (Schneider et al., 2012) was used for determining the signal strength of *CmNRT* and CmMYB1 from **(D,E)**. Transcript accumulation of *CmNRT* was calculated by dividing the strength of *CmNRT* transcript levels into the strength of CmMYB1 protein levels. Values represent the average transcript accumulation of *CmNRT* in three independent experiments. Error bars indicate the standard deviation (SD). Significant differences were determined using one-way ANOVA (for the +N condition, *p* = 0.0000012; for the −N condition, *p* = 0.000056) followed by *post hoc* tests. The asterisks denote the difference between the “pPD1–523/ + N” sample *vs.* other +N condition samples, the “pPD1–523/−N” sample *vs.* other −N condition samples, respectively (***p ≤ 0.001, **p ≤ 0.01, n.s., *p* > 0.0125). **(G)** Quantitative real-time PCR (qRT-PCR) analysis of the transcript accumulation of nitrogen assimilation genes. Of the total RNAs per condition of pPD1–310 and pPD1–523 from **(D)**, 500 ng was used for genome DNA re-movement and cDNA synthesis. Results were from three independent experiments, and data represent transcript accumulation of each gene. Asterisks indicate the statistical significance of differences between the pPD1–310/+N sample and the pPD1–523/+N sample, the pPD1–310/−N sample and the pPD1–523/−N sample, respectively (*p < 0.05, **p < 0.01, ***p < 0.001, ****p < 0.0001, n.s., *p* > 0.05; Student’s *T*-test).

Functions of TFs are usually modulated by their regulatory elements that bind to cofactors and metabolite molecules (Xie et al., 2006; Puga et al., 2014; Le et al., 2016; Qi et al., 2017; Zhu et al., 2019; Wang et al., 2020) or by post-translational modifications (Gill, 2003; Tootle and Rebay, 2005; Schütze et al., 2008). Therefore, we hypothesized that the underlying mechanism related to nitrogen status-dependent CmMYB1 function could be revealed by the identification of the CmMYB1 regulatory element. To test this hypothesis, using a non-*CmMYB1* background strain combined with a plasmid series expressing partially truncated CmMYB1s would be a good strategy. Since CmMYB1 would only be expressed exogenously; different phenotypes among the strains must be caused by the different CmMYB1 sequences. Firstly, we designed and obtained a *CmMYB1* knockout strain named BZ1 (Supplementary Figure 1), which shows uracil auxotrophy and 5-FOA resistance (Figures 1A,B) as a host strain for the analysis. We constructed a series of plasmids expressing FLAG-fused CmMYB1 in different amino acid sequence patterns from the carboxyl-terminus, which is regulated by *CmMYB1* own promoter. After transforming each plasmid into BZ1, we succeeded in obtaining all five types of CmMYB1 transformants (Supplementary Figures 2, 3): pPD1–310, which expresses +1 to +310 amino acids of CmMYB1 (+1 indicates the start position of translation), pPD1–380, pPD1–460, pPD1–99, 201–523, and pPD1–523 (Figure 1C).

A previous study showed that the *CmNRT* transcript was completely CmMYB1 dependent. It was increased under −N conditions and came to a peak at the fourth hour after −N treatment (Imamura et al., 2009). Thus, the transcript level of *CmNRT* at the fourth hour after −N could well represent the functional status of CmMYB1 in the cell. We first investigated the transcripts before (+N) and after the −N condition in each constructed strain by Northern blot analysis (Figure 1D). We evaluated *CmNRT* transcript levels after normalizing with CmMYB1 protein levels in each strain since the CmMYB1 protein level could affect the transcripts. Our recent work has demonstrated that overexpressing *CmMYB1* in *C. merolae* significantly increased *CmNRT* transcripts under the +N condition (Zhou et al., 2021). To monitor CmMYB1 protein levels, we conducted dot blot analysis since different sizes of proteins could cause distinct membrane transfer efficiencies during immunoblot analysis (Figure 1E and Supplementary Figure 3). Eventually, we got the transcript accumulation of *CmNRT*, and the results showed that the *CmNRT* transcript level was higher in pPD1–310 than in pPD1–523 under the +N condition (Figure 1F). No *CmNRT* transcripts were observed in pPD1–380, pPD1–460, or pPD1–99, 201–523, same as in pPD1–523 (Figure 1D). On the contrary, *CmNRT* transcripts showed no difference in pPD1–310 as opposed to pPD1–523 under the −N condition, whereas it was lower in pPD1–380, pPD1–460, and pPD1–99, 201–523 (Figure 1F). These results clearly suggested that +311 to +380 amino acids of CmMYB1 negatively regulate the transcripts of *CmNRT* under the +N condition.

Next, we wondered whether this regulation could also be observed in other nitrogen assimilation genes expression. Therefore, we checked these transcripts under the same condition shown in Figure 1D by quantitative real-time PCR (qRT-PCR) analysis using specific primer sets (Supplementary Table 2). The results showed that transcript accumulation of nitrate assimilation genes, *CmNRT*, *CmNR*, and *CmNIR* (encoding nitrite reductase, NIR), was higher in pPD1–310 but lower or no change in pPD1–380, pPD1–460, and pPD1–99, 201–523 than in pPD1–523 under the +N condition (Figure 1G and Supplementary Figures 4A–C). In the case of the transcript accumulation of *CmNRT* and *CmNR* under the −N condition, a significant decrease was observed in pPD1–380, pPD1–99, 201–523 compared with pPD1–523, whereas there was no difference in pPD1–310 (Figure 1G and Supplementary Figures 4A,B). Contrarily, transcript accumulation of *CmAMT* and *CmGS* was lower in pPD1–310 and other transformants than in pPD1–523 under + N and −N conditions, respectively (Figure 1G and Supplementary Figures 4D,E). These data clearly revealed that the +311 to +380 amino acid region of CmMYB1 functions as a negative domain for CmMYB1 (hereafter ND) and specifically regulates transcription of nitrate assimilation genes under the +N condition.

### Effects of Subcellular Localization and the Promoter-Binding Ratio of CmMYB1 by Negative Domain

**FIGURE 2:**
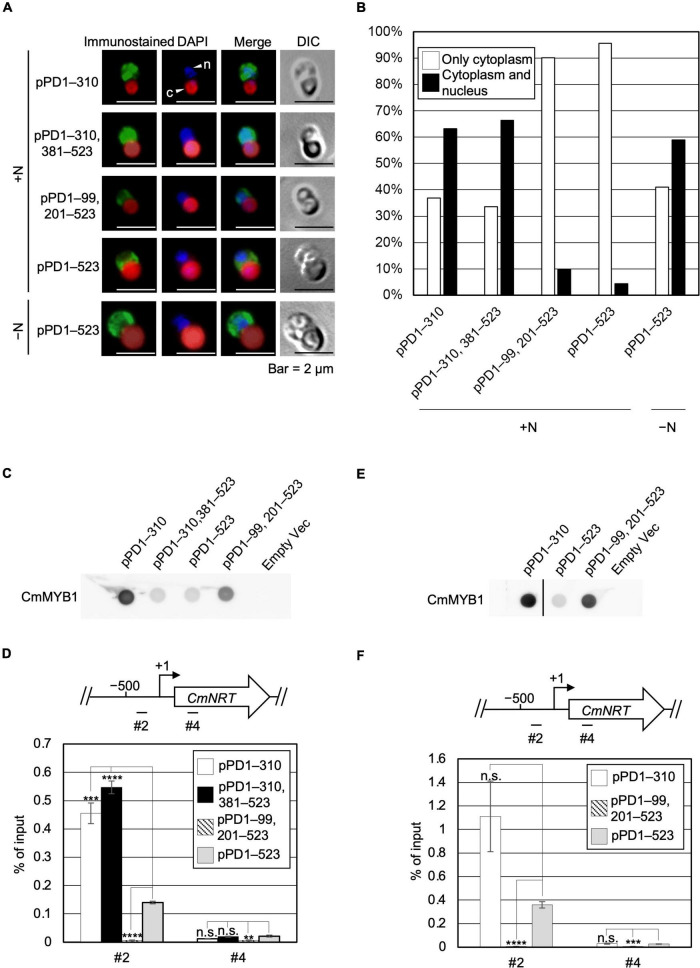
Subcellular localization and a promoter-binding ratio of CmMYB1 under the +N/−N conditions. **(A)** Subcellular localization of CmMYB1 in CmMYB1 partially truncated transformants. Fixed cells under the +N/−N conditions for 4 h were reacted with an anti-FLAG antibody (Wako, Osaka, Japan), and the localization of FLAG-fused CmMYB1 was detected with an Alexa Fluor 488-conjugated donkey anti-mouse antibody (Thermo Fisher Scientific, Waltham, MA, United States, a green signal from “immunostained” pictures). DAPI staining of nucleus DNA (a blue signal from “DAPI” pictures), merged image of immunostaining and DAPI staining (Merge), and differential interference contrast image (DIC) are shown. Positions of nucleus (n) and chloroplast (c) were indicated with arrowheads (a scale bar, 2 μm). **(B)** Statistical analysis of CmMYB1 localization in CmMYB1 partially truncated transformants. Over 110 cells per strain under the +N condition were used in this analysis. Photos of fluorescence of CmMYB1 and the nucleus were treated with the same parameter, and colocalization of CmMYB1 with the nucleus was determined by covering CmMYB1 fluorescence with the nucleus fluorescence. **(C)** Protein levels of FLAG-fused CmMYB1 under the +N condition in CmMYB1 partially truncated transformants. Aliquots containing 16.6 μg of total proteins from the *C. merolae* cells harvested under the +N condition for 4 h were dropped on a hydrophilized PVDF membrane and analyzed with an anti-FLAG antibody (Wako, Osaka, Japan). Proteins extracted from empty vector transformants (Empty Vec) were used as a negative control. Same experiments were performed three times, independently. **(D)** Determination of the CmMYB1 promoter-binding ratio to *CmNRT in vivo* under the +N condition. CmMYB1 partially truncated transformant cells were treated under the +N condition for 4 h and subsequently fixed for ChIP analysis. The schematic diagram above the image indicates the analyzed gene *CmNRT* and positions that were amplified by qRT-PCR following ChIP (+1 indicates the transcription start site). The anti-FLAG antibody (Wako, Osaka, Japan) was used in this ChIP analysis. Values are averages of three independent experiments, and % of input was calculated by dividing percentage recovery from ChIP analysis into CmMYB1 protein levels. Error bars indicate the standard deviation (SD). Significant differences were determined using one-way ANOVA (for position #2 of *CmNRT*, *p* = 0.00000029; for position #4 of *CmNRT*, *p* = 0.0034), followed by *post hoc* tests. The asterisks denote the difference between the pPD1–523 +N sample vs. the other samples in position #2 or #4 of *CmNRT*, respectively. (****p ≤ 0.0001, ***p ≤ 0.001, **p ≤ 0.01, n.s., *p* > 0.0167). **(E)** Protein levels of FLAG-fused CmMYB1 under the −N condition in CmMYB1 partially truncated transformants. Aliquots containing 16.6 μg of total proteins from the *C. merolae* cells harvested under the −N condition for 4 h were dropped on a hydrophilized PVDF membrane and analyzed with an anti-FLAG antibody (Wako, Osaka, Japan). Proteins extracted from empty vector transformants (Empty Vec) were used as a negative control. The pPD1–310 +N lane and the right membrane are on the same membrane. A straight line was added between the pPD1–310 +N lane and the pPD1–523 +N lane since we removed some additional signal between these two lanes. Same experiments were performed three times, independently. **(F)** Determination of the CmMYB1 promoter-binding ratio to *CmNRT in vivo* under the −N condition. CmMYB1 partially truncated transformant cells were treated under the −N condition for 4 h and subsequently fixed for ChIP analysis. The schematic diagram above the image indicates the analyzed gene *CmNRT* and positions that were amplified by qRT-PCR following ChIP (+1 indicates the transcription start site). The anti-FLAG antibody was used in this ChIP analysis. Values are averages of three independent experiments, and % of input was calculated by dividing percentage recovery from ChIP analysis into CmMYB1 protein levels. Error bars indicate the standard deviation (SD). Significant differences were determined using one-way ANOVA (for position #2 of *CmNRT*, *p* = 0.0021; for position #4 of *CmNRT*, *p* = 0.000059), followed by *post hoc* tests. The asterisks denote the difference between the pPD1–523 −N sample *vs.* the other samples in position #2 or #4 of *CmNRT*, respectively. (****p ≤ 0.0001, ***p ≤ 0.001, n.s., *p* > 0.025).

Given the evidence that ND negatively regulates transcription of nitrate assimilation genes under the +N condition, we considered two possibilities regarding the regulation, namely localization and the DNA-binding ratio of CmMYB1. We first investigated the effect of ND on the intracellular localization of CmMYB1 by immunostaining analysis before (+N) and after the −N condition. In addition to the strains used in Figure 1, to investigate the impact of only the ND region, we further constructed a transformant expressing +1 to +310, +381 to +523 amino acids of CmMYB1 and named it pPD1–310, 381–523 (Supplementary Figure 2). The results showed that CmMYB1 in pPD1–523 and pPD1–99, 201–523 was localized in the cytoplasm under the +N condition (Figure 2A). Contrarily, ND truncated CmMYB1 in pPD1–310, 381–523, and pPD1–310 showed nucleus and cytoplasm localizations that were similarly observed in pPD1–523 under the −N condition (Figure 2A). Besides, localization data from over 110 cells per strain under the +N/−N condition were subjected to statistical analysis. Results showed that CmMYB1 in about 4% of cells was localized in the cytoplasm and nucleus in pPD1–523 under the +N condition, whereas CmMYB1 in over 63% of cells exhibited the same distribution in pPD1–310 and pPD1–310, 381–523 (Figure 2B). On the other hand, CmMYB1 in about 58% of cells was localized in the cytoplasm and nucleus in pPD1–523 under the −N condition. Results of immunostaining analysis and statistical analysis implied that ND maintains CmMYB1 in the cytoplasm under the +N condition.

Next, we checked the other possibility, namely, the effect of ND on the DNA-binding efficiency of CmMYB1. The previous study has shown that CmMYB1 specifically binds to the proximal promoter region of *CmNRT* under the −N condition but not the distinct region (Imamura et al., 2009). Therefore, we tested the promoter-binding ratio of several truncated CmMYB1s at these two regions under the +N condition through chromatin immunoprecipitation (ChIP) analysis. We normalized the values from ChIP analysis with CmMYB1 protein levels from dot blot analysis (Figure 2C). The results showed that ND-truncated CmMYB1 in pPD1–310, 381–523, and pPD1–310 exhibited a specifically higher promoter-binding ratio to the #2 region of *CmNRT* (Figure 2D, up panel) than the full length of CmMYB1 in pPD1–523 under the +N condition (Figure 2D). In contrast, the similar high-binding ratio of CmMYB1 detected in the #2 region was not observed in the #4 region (Figure 2D). We also performed ChIP analysis under the −N condition using pPD1–310, pPD1–99, 201–523, and pPD1–523. We normalized the values from ChIP analysis with CmMYB1 protein levels from dot blot analysis (Figure 2E). The results showed that the promoter-binding ratio to the #2 region of *CmNRT* showed no difference between ND-truncated CmMYB1 in pPD1–310 and the full length of CmMYB1 in pPD1–523 (Figure 2F). These results exhibited that ND inhibits the promoter-binding ratio of CmMYB1 under the +N condition, but not the −N condition.

### Identification of Negative Domain-Interacting Proteins

**FIGURE 3:**
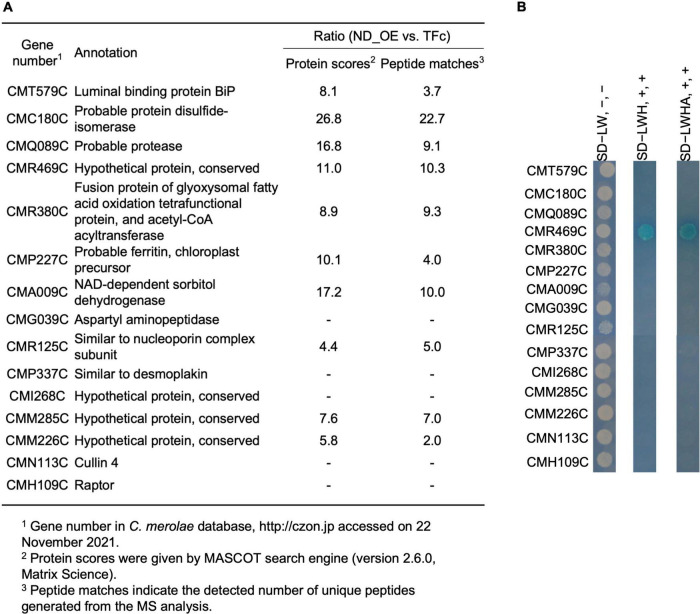
A list of ND-binding proteins from LC-MS/MS analysis and confirmation of ND-binding proteins by yeast two-hybrid analysis. **(A)** A list of potential ND-binding proteins. ND-overexpressing and control samples were used in LC-MS/MS analysis. The ND-binding protein list was conducted depending on the rate of protein scores and the rate of peptide matches. The proteins whose protein scores ratio and peptide matches ratio were both below 5.0. were cut off, and the remaining top 15 candidates were listed up. “–” denotes that a signal from the control sample was not detected. **(B)** Confirmation of ND-binding proteins in yeast. DNA sequence of the ND region was used to construct BD plasmid. Each ORF of the top 15 candidates from LC-MS/MS analysis was used to construct AD plasmids. Each AD plasmid was cotransformed into yeast gold competent cells with BD plasmid, respectively, and spread on leucine (L) and tryptophan (W)-lacking (SD−LW) plates. Resulted transformants were further diluted and spotted on SD−LW plates without adding 5-Bromo-4-chloro-3-indolyl-α-D-galactopyranoside [X-α-Gal], [Wako, Osaka, Japan] and Aureobasidin A [AbA], [Takara, Shiga, Japan] (SD−LW, -, -, left lane), L, W, and histidine (H) lacking but with X-α-Gal and AbA plates (SD−LWH, +, +, middle lane), and L, W, H and alanine (A) lacking but with X-α-Gal and AbA plates (SD−LWHA, +, +, right lane), and incubated at 30°C for 1 day (SD−LW plates) or 1 week (SD−LWH, SD−LWHA plates) prior to photography.

Since ND regulates CmMYB1 localization and the promoter-binding ratio under the +N condition, we decided to further investigate the underlying mechanism. We assumed ND is controlled by its binding protein(s), because, in *Saccharomyces cerevisiae*, transcriptional regulator Ure2 binds to Gln3 and maintains Gln3 in the cytoplasm under the +N condition (Blinder et al., 1996; Beck and Hall, 1999; Carvalho and Zheng, 2003). To investigate the possibility, we first constructed a FLAG-fused ND-overexpressing strain (ND_OE) and its control strain (TFc) (Supplementary Figure 5). We performed a pull-down assay using ND_OE and TFc grown under the +N condition, and the resultant pull-down products were subjected to LC-MS/MS. After analyzing the data, we eventually obtained a list of potential ND-interacting proteins (Supplementary Table 3). Among them, based on the setting rule that the protein score is higher than 130, we picked up the top 15 candidates for further analysis (Figure 3A).

To confirm the interaction between ND and those candidates, we performed a yeast two-hybrid (Y2H) analysis. Results showed that transformants coexpressing ND and CMR469C (protein number in the *C. merolae* database^2^), annotated as a hypothetical protein with a comment as a “cyanobacterial protein,” could grow on SD−LWH media and SD−LWHA, stronger selection media than SD−LWH (Figure 3B). These data, together with the pull-down assay, showed that CMR469C interacts with the ND of CmMYB1. We named CMR469C as CmNDB1, short for *C. merolae* Negative Domain Binding Protein 1.

### Effect of Subcellular Localization and the Promoter-Binding Ratio of CmMYB1 by Deletion of *CmNDB1*

Based on the results observed from the Y2H analysis, we investigated whether CmNDB1 interacts with CmMYB1 in the *C. merolae* cell. Thus, we investigated the possibility by co-immunoprecipitation (Co-IP) analysis using a strain in which Myc-fused CmNDB1 and FLAG-fused CmMYB1 were expressed. We could not observe the interaction between CmNDB1 and CmMYB1 in *C. merolae* cells under many experimental conditions (see section “Discussion”).

**FIGURE 4:**
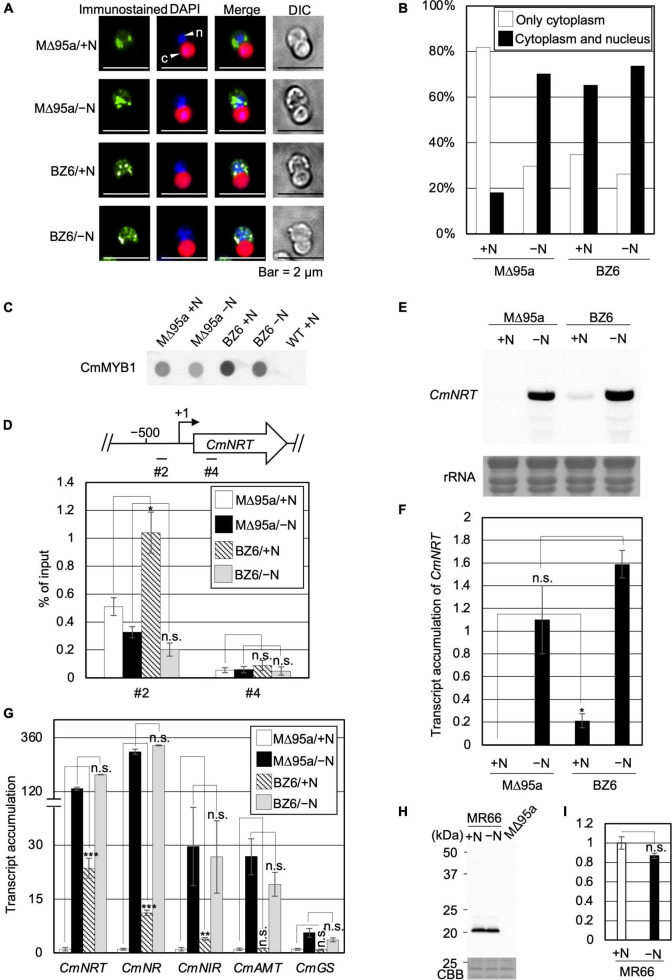
Subcellular localization, the promoter-binding ratio of CmMYB1 and expression of nitrogen-assimilation genes in MΔ95a and BZ6 under the +N/−N conditions. **(A)** Subcellular localization of CmMYB1 in MΔ95a and BZ6 cells under the +N/−N conditions. Cells were fixed and reacted with an anti-FLAG antibody (Wako, Osaka, Japan), and the localization of FLAG-fused CmMYB1 was detected with an Alexa Fluor 488-conjugated donkey anti-mouse antibody (Thermo Fisher Scientific, Waltham, MA, United States, a green signal from “immunostained” pictures). DAPI staining of nucleus DNA (a blue signal from “DAPI” pictures), merged image of immunostaining and DAPI staining (Merge), and differential interference contrast image (DIC) are shown. Positions of the nucleus (n) and chloroplast (c) were indicated with arrowheads (a scale bar, 2 μm). **(B)** Statistical analysis of CmMYB1 localization in MΔ95a and BZ6 cells treated under +N/−N for 4 h. Over 110 cells per strain under the +N/−N conditions were used in this analysis. Photos of fluorescence of CmMYB1 and the nucleus were treated with the same parameter, and colocalization of CmMYB1 with the nucleus was determined by covering CmMYB1 fluorescence with the nucleus fluorescence. **(C)** Protein levels of FLAG-fused CmMYB1 in MΔ95a and BZ6 under the +N/−N conditions. Aliquots containing 16.6 μg of total proteins from the *C. merolae* cells harvested under the same condition as in **(A)** were dropped on a hydrophilized PVDF membrane and analyzed with an anti-FLAG antibody (Wako, Osaka, Japan). Proteins extracted from WT were used as a negative control. Same experiments were performed 3 times, independently. **(D)** Determination of the CmMYB1 promoter-binding ratio to *CmNRT in vivo*. MΔ95a and BZ6 cells were treated under the +N/−N conditions for 4 h as in **(A)**, and the cells were subsequently fixed for ChIP analysis. The schematic diagram above the image indicates the analyzed gene *CmNRT* and positions that were amplified by qRT-PCR following ChIP (+1 indicates the transcription start site). The anti-FLAG antibody was used in this ChIP analysis. Values are averages of three independent experiments, and % of input was calculated by dividing percentage recovery from ChIP analysis into CmMYB1 protein levels. Error bars indicate the standard deviation (SD). Asterisks indicate the statistical significance of differences between the MΔ95a + N sample *vs.* the BZ6+N sample, the MΔ95a −N sample *vs.* the BZ6 −N sample in position #2 or #4 of *CmNRT*, respectively (*p < 0.05, n.s., *p* > 0.05; Student’s *T*-test). **(E)** Transcript levels of *CmNRT* in MΔ95a and BZ6 under the +N/−N conditions. Cells treated under +N/−N for 4 h were harvested, and total RNAs were prepared from the cells. Northern blot analysis was performed with total RNAs (3 μg) using a specific probe for *CmNRT*. The bottom panel shows rRNA stained with methylene blue as a loading control. **(F)** Transcript accumulation of *CmNRT* in MΔ95a and BZ6. The signal strength of *CmNRT* and CmMYB1 from **(C,E)** was determined by ImageJ, same as in Figure 1F, respectively. Transcript accumulation of *CmNRT* was calculated by dividing the strength of *CmNRT* transcript levels into the strength of CmMYB1 protein levels. Values represent the average transcript accumulation of *CmNRT* in three independent experiments. Error bars indicate the standard deviation (SD). Asterisks indicate the statistical significance of differences between the +N samples (*p < 0.05, n.s., *p* > 0.05; Student’s *T*-test). **(G)** qRT-PCR analysis of the transcript accumulation of nitrogen assimilation genes. Of the total RNAs per condition of MΔ95a and BZ6 from **(E)**, 500 ng was used for genome DNA removement and cDNA synthesis. Results were from three independent experiments, and data represent transcript accumulation of each gene. Asterisks indicate the statistical significance of differences between the MΔ95a/+N sample *vs*. the BZ6/+N sample, the MΔ95a/−N sample *vs.* the BZ6/−N sample, respectively (**p < 0.01, ***p < 0.001, n.s., *p* > 0.05; Student’s *T*-test). **(H)** Protein levels of CmNDB1 under the +N/−N conditions. Aliquots containing 12 μg of total proteins from the *C. merolae* cells harvested under the +N/−N conditions were separated by 12% SDS-PAGE and analyzed by immunoblot analysis with an anti-Myc antibody (MBL, Tokyo, Japan). Protein extracted from the MΔ95a cells was used as a negative control. The positions of molecular size markers are indicated in kilodaltons (kDa) at the left. Same experiments were performed 3 times, independently. **(I)** Statistical analysis of CmNDB1 protein levels. Data from **(H)** were used in this analysis. n.s., *p* > 0.05; Student’s *T*-test.

Therefore, we tried to reveal the effect of CmNDB1 on the regulation of CmMYB1 functions through the genetic analysis approach. To examine this, we first constructed a strain named WM1 (Supplementary Figure 6), in which FLAG-fused CmMYB1 was expressed. Using WM1, we then obtained a strain named MΔ95a (Supplementary Figure 7), which was used as a parent strain for constructing a *CmNDB1* knockout strain named BZ6 (Supplementary Figure 8). We investigated CmMYB1 localization by immunostaining analysis before (+N) and after the −N condition using MΔ95a and BZ6. The results showed that CmMYB1 was localized in the cytoplasm in MΔ95a, a parent strain of BZ6, whereas it was localized in both the cytoplasm and nucleus in BZ6 under the +N condition (Figure 4A, a green signal from the first and third rows). Contrarily, CmMYB1 exhibited cytoplasm and nucleus distribution in both MΔ95a and BZ6 under the −N condition (Figure 4A, a green signal from the second and fourth rows). Besides, localization data from over 110 cells per strain under the +N or −N condition were subjected to statistical analysis. Results showed that CmMYB1 in about 18% of cells was localized in the cytoplasm and nucleus in MΔ95a under the +N condition, whereas CmMYB1 in over 65% of cells exhibited the same distribution in BZ6 (Figure 4B). On the contrary, cytoplasm and nucleus localization of CmMYB1 was observed in over 70% MΔ95a cells, 73% BZ6 cells, under the −N condition, respectively (Figure 4B). Results of immunostaining analysis and statistical analysis indicated that CmNDB1 contributes to maintaining CmMYB1 in the cytoplasm under the +N condition.

Since localization of CmMYB1 was altered in the *CmNDB1* deletion strain, we wondered whether the promoter-binding ratio of CmMYB1 was also influenced by deleting *CmNDB1*. To this end, we examined this possibility by ChIP analysis and normalized the values with CmMYB1 protein levels from dot blot analysis (Figure 4C). Results showed that CmMYB1 exhibited a specifically higher promoter-binding ratio to the #2 region of *CmNRT* in BZ6 than in MΔ95a under the +N condition (Figure 4D). In contrast, the promoter-binding ratio of CmMYB1 to the #2 region showed no difference under the −N condition between the two strains (Figure 4D). In both strains, reduced promoter-binding ratios of CmMYB1 were observed under the −N condition than under the +N condition, which is inconsistent with our previous result observed in WT (Imamura et al., 2009). The reason that caused these different change patterns of CmMYB1 promoter-binding ratios between WT and MΔ95a is still unknown. One possibility could be the chromatin architecture change around *CmMYB1* due to the insertion of construction for FLAG epitope tag expression. These results suggested that CmNDB1 reduces the CmMYB1 promoter-binding ratio under the +N condition.

### Effect on Transcription of Nitrate Assimilation Genes by Deletion of *CmNDB1*

Subcellular localization and ChIP analysis data above showed that *CmNDB1* deletion caused cytoplasmic and nuclear localization of CmMYB1 and a higher promoter-binding ratio of CmMYB1 under the +N condition. Does *CmNDB1* deletion also influence the transcription of nitrogen assimilation genes? To answer this, we first investigated the *CmNRT* transcripts in MΔ95a and BZ6 (Figure 4E) by Northern blot analysis. We normalized *CmNRT* transcript levels using CmMYB1 protein levels (Figure 4C). The result showed that the transcript accumulation of *CmNRT* was higher in BZ6 than in MΔ95a under the +N condition, whereas there was no difference under the −N condition (Figure 4F). Furthermore, we examined the *CmNRT* transcripts by adding ammonium into MΔ95a and BZ6 cells after −N treatment. Results showed that *CmNRT* transcripts in BZ6 reduced to 17.3 and 7.5% after adding ammonium for 0.5 and 1 h, respectively (Supplementary Figure 9). A much more severe reduction of *CmNRT* transcripts was observed in MΔ95a (0.5 and 0% at the indicated time shown above) (Supplementary Figure 9). These results indicated that *CmNDB1* deletion increases *CmNRT* transcripts under the +N condition.

Next, we checked the transcripts of other nitrogen assimilation genes under the same condition shown in Figure 4E by qRT-PCR analysis. The results showed that transcript accumulation of nitrate assimilation genes, *CmNRT*, *CmNR*, and *CmNIR*, was significantly increased in BZ6 against in MΔ95a under the +N condition (Figure 4G). Contrarily, the transcript levels of *CmAMT* and *CmGS* showed no difference between the two strains (Figure 4G). In the case of the −N condition, transcript accumulation of all nitrogen assimilation genes showed no significant difference between the two strains (Figure 4G). Altogether, these results clearly showed that *CmNDB1* deletion specifically increases the transcription of nitrate assimilation genes under the +N condition.

### The Expression Level of CmNDB1 Under the +N/−N Condition

Finally, we investigated the protein expression level of CmNDB1 under the +N/−N condition. To achieve this, we constructed a Myc-tagged CmNDB1 strain, MR66 (Supplementary Figure 10), using MΔ95a as the host strain. The result of immunoblot analysis using proteins extracted from MR66 cells under the +N/−N condition for 4 h showed that CmNDB1 exhibited no significant change between the +N and −N conditions (Figures 4H,I). The immunoblot analysis result indicated that CmNDB1 regulates the function of CmMYB1 without changing the CmNDB1 protein level.

## Discussion

**FIGURE 5:**
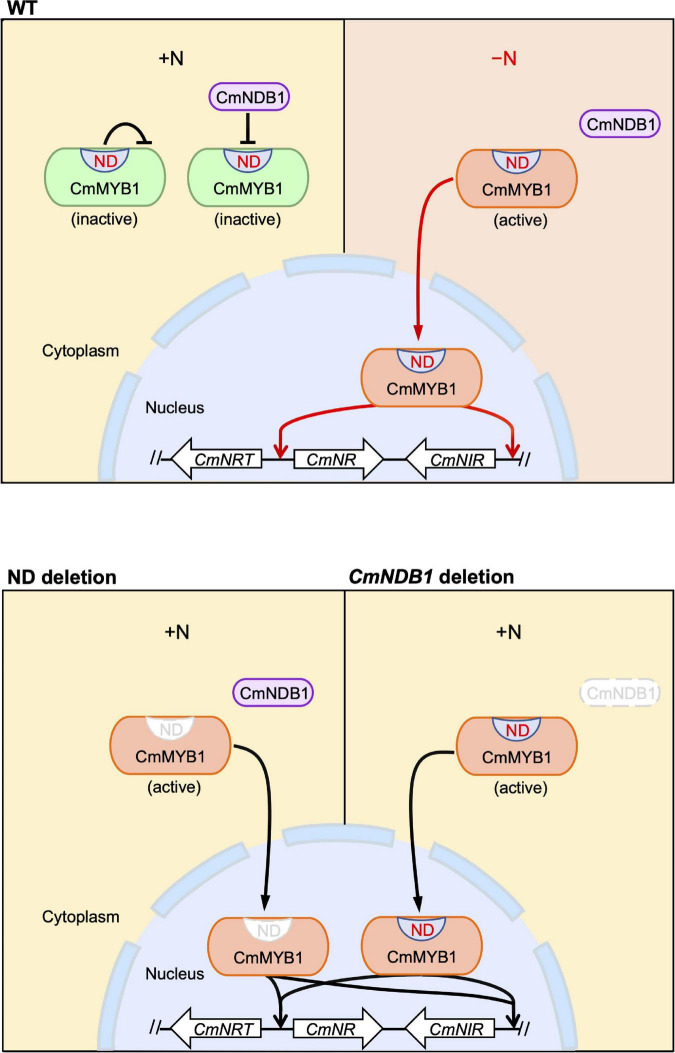
A predicted model of an activation mechanism of CmMYB1 in *C. merolae*. “ND” is short for negative domain of CmMYB1. In case of WT, under the +N condition, ND and CmNDB1 maintain CmMYB1 in the cytoplasm and repress its ability of induction of CmMYB1-dependent transcription. Under the −N condition, repression from ND and CmNDB1 is resolved, which results in nucleus localization of CmMYB1. Finally, CmMYB1 specifically induces the transcription of nitrate assimilation genes. In case of ND or *CmNDB1* deletion, under the +N condition, CmMYB1 is translocated from cytoplasm to nucleus, where it starts to induce the transcription of nitrate assimilation genes.

Given the evidence shown above, we proposed a working model of the regulatory mechanism of CmMYB1 in *C. merolae* (Figure 5). The ND and CmNDB1 contribute to maintaining CmMYB1 in the cytoplasm (Figures 2A,B, 4A,B) under the +N condition. The ND or *CmNDB1* deletion results in CmMYB1 nuclear localization and an increased CmMYB1 promoter-binding ratio (Figures 2D, 4D), which, in turn, induces transcription of nitrate assimilation genes (Figures 1F,G, 4F,G). The ND may function synergistically with CmNDB1 due to two observations: one is the interaction between two proteins from the Y2H analysis; the other is the same upregulation pattern of nitrate assimilation genes transcription in ND or *CmNDB1* deletion transformant under the +N condition.

Since CmNDB1 interacts with ND of CmMYB1 in Y2H analysis (Figure 3B), we investigated the interaction between CmNDB1 and CmMYB1 in the cells by co-IP analysis. Besides, we applied dithiobis [succinimidyl propionate] (DSP), a membrane-permeable crosslinker, to fix the interaction between protein complexes. However, we could not get any positive binding evidence from both analyses at least under the examined conditions so far. One possibility is that the interaction between CmMYB1 and CmNDB1 is not rigid and hard to be monitored by IP in the cells. The other possibility that must be considered is post-translational modifications of CmMYB1 and/or CmNDB1. Our previous study showed that the target of rapamycin (TOR) inhibition resulted in increased transcripts of nitrate assimilation genes under the +N condition (Imamura et al., 2015), implying that phosphorylation of CmMYB1 or its partner protein(s) is involved in regulating transcription of nitrate assimilation genes under the +N condition. In rice, it has been revealed that exogenous Cry1Ab/c protein binds to endogenous CP43 or DnaJ in Y2H analysis. Although a weak interaction was observed between Cry1Ab/c and CP43 in bimolecular fluorescence complementation analysis, no interaction between these proteins was observed in co-IP analysis in the cells (Fu and Liu, 2020). This observed difference may be caused by the detection limitations of these technologies or could also be possible due to the ubiquitination of DnaJ (Zhong et al., 2018). Similarly, investigation of the post-translational modifications of CmMYB1 and/or CmNDB1 for their interaction in the cells would be required to solve the regulation mechanism between those proteins *in vivo* as a future project.

Our previous studies have shown that transcription of nitrate assimilation genes is regulated by the CmMYB1-dependent nitrogen catabolite repression (NCR) mechanism (Imamura et al., 2009, 2010). Transcripts of *CmNRT*, *CmNR*, and *CmNIR* were increased after shifting from an ammonium medium to a nitrate medium. This increase was completely abolished in the *CmMYB1* null strain (Imamura et al., 2010). In the present study, increased transcripts of the nitrate assimilation genes were observed from ND or *CmNDB1* deletion mutants cultured in the ammonium medium (Figures 1F,G, 4F,G), where CmMYB1-dependent NCR was working. This raises the possibility that CmNDB1 and/or ND are involved in the NCR mechanism. Interestingly, deletion of ND or *CmNDB1* did not result in an increase of *CmAMT* and *CmGS* transcripts under the +N condition (Figures 1G, 4G). This could be due to the predominant role of additional regulators, including TF(s) other than CmMYB1 for *CmAMT* and *CmGS* transcripts since these transcripts were still presented in the *CmMYB1* null strain under the +N condition (Imamura et al., 2009). The other possibility could be the different promoter architectures between nitrate assimilation genes and *CmAMT* and *CmGS*. The nitrate assimilation genes are located next to each other and form clusters on the chromosome (Imamura et al., 2010), suggesting that the expression of nitrate assimilation genes could be controlled by identical regulators, such as CmNDB1, and regulatory pathway(s) that are not involved in C*mAMT* and *CmGS* transcription. Genome-wide effect/regulation of CmMYB1 by ND and CmNDB1 needs further research.

The ND or *CmNDB1* deletion resulted in CmMYB1 localization change (Figures 2A,B, 4A,B). How do these two factors regulate the localization under the +N condition? One possibility could be the post-translational phosphorylation of ND. Phosphorylation prediction analysis using NETPhos 3.1 (Blom et al., 2004) reported 56 sites among 523 amino acids of CmMYB1 (10.7%) that were predicted to be phosphorylated. Interestingly, phosphorylation sites were highly enriched in ND. Among 70 amino acids of ND, 18 sites (25.7%) were predicted to be phosphorylated (Supplementary Figure 11). The MS analysis that we performed supported the prediction since we detected 12 phosphorylation sites among NDs under the +N condition (Supplementary Figure 11). In *Saccharomyces cerevisiae*, it has been revealed that Gln3 was phosphorylated under the +N condition (Cox et al., 2004). A recent study has shown that Gln3 nuclear localization was abolished by substituting serine to aspartate in the sites of +505 to +594 (Tate et al., 2021). Further investigation focusing on *in vivo* phosphorylation modification would help in understanding the underlying mechanism of CmMYB1 localization changes.

## Data Availability Statement

The datasets presented in this study can be found in online repositories. The names of the repository/repositories and accession number(s) can be found in the article/Supplementary Material.

## Author Contributions

BZ and SI designed the research. BZ, HS, and SI performed the research and wrote the manuscript. BZ, HS, KI, KT, and SI analyzed the data. All the authors contributed to the article and approved the submitted version.

## Conflict of Interest

SI was employed by Nippon Telegraph and Telephone Corporation. The remaining authors declare that the research was conducted in the absence of any commercial or financial relationships that could be construed as a potential conflict of interest.

## Publisher’s Note

All claims expressed in this article are solely those of the authors and do not necessarily represent those of their affiliated organizations, or those of the publisher, the editors and the reviewers. Any product that may be evaluated in this article, or claim that may be made by its manufacturer, is not guaranteed or endorsed by the publisher.

## Footnotes

1
http://merolae.biol.s.u-tokyo.ac.jp/download/orf.fasta

2
http://czon.jp
