# Supplementary material for: CmNDB1 and a Specific Domain of CmMYB1 Negatively Regulate CmMYB1-Dependent Transcription of Nitrate Assimilation Genes Under Nitrogen-Repleted Condition in a Unicellular Red Alga
Source: Front Plant Sci. 2022 Mar 11;13:821947. doi: 10.3389/fpls.2022.821947 (PMC8962646; doi:10.3389/fpls.2022.821947)
Supplement: Supplementary file 1 [file Presentation_1.PDF]

A

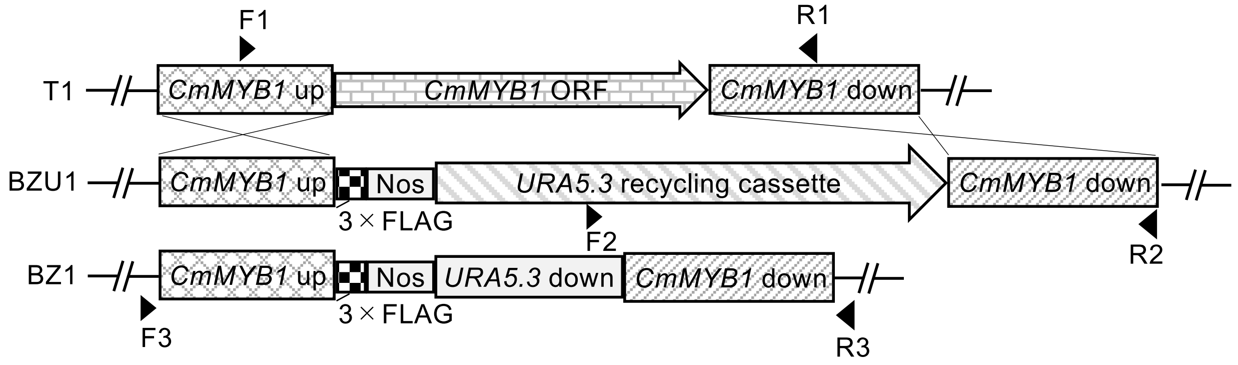

B

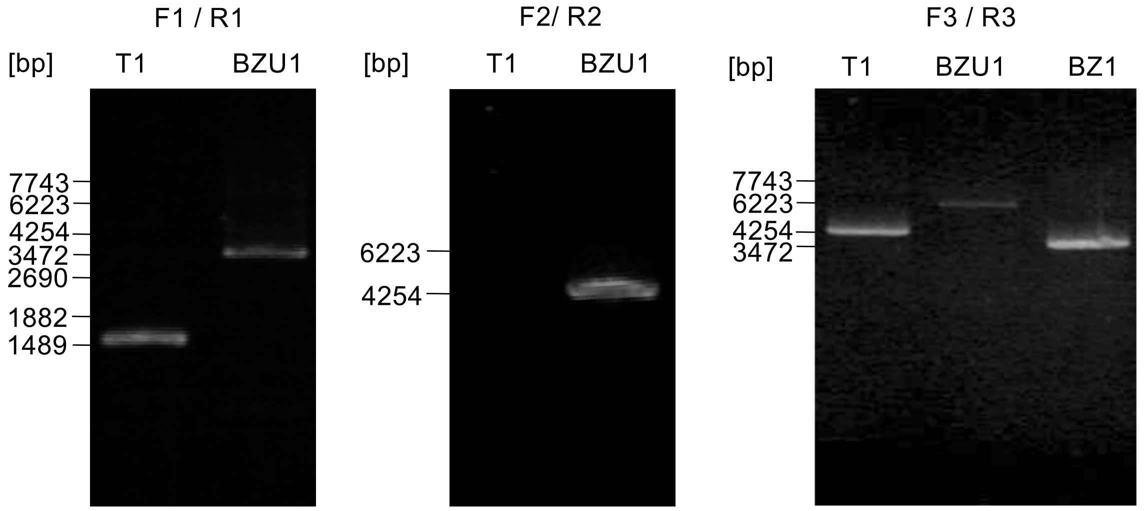

Supplementary Figure 1. Acquisition of *CmMYB1* knock out strain, BZU1 and BZ1. **(A)** Schematic representation of the *CmMYB1* ORF locus in the T1 (top panel), BZU1 (middle panel), and BZ1 (bottom panel) strains. "*CmMYB1* up" and "*CmMYB1* down" indicate 1500-bp upstream and downstream regions of *CmMYB1*, respectively, used for homologous recombination. "*CmMYB1* ORF" indicates the 1569-bp open reading frame of *CmMYB1*. "Uracil recycling cassette" indicates a 3234-bp fragment used for uracil auxotrophic transformant selection. "*URA5.3* down" indicates 483-bp downstream regions of *URA5.3*. The positions of primers for PCR are shown with arrowheads. **(B)** Confirmation of the transformation. The genomic DNAs from T1, BZU1, and BZ1 were analyzed by PCR with a set of primers (listed in Table S1), F1/R1 (Left), F2/R2 (Middle), or F3/R3 (Right). The PCR products were resolved by 0.8% agarose gel electrophoresis. The positions of a molecular size marker are indicated as bp at the left. The size of each PCR product detected is shown below as predicted. T1 group: 1619 bp, BZU1 group: 3689 bp (Left); T1 group: no purification, BZU1 group: 4273 bp (Middle); T1 group: 4626 bp, BZU1 group: 6696 bp, BZ1 group: 3483 bp (Right).

Zhou et al., Supplementary Figure 2

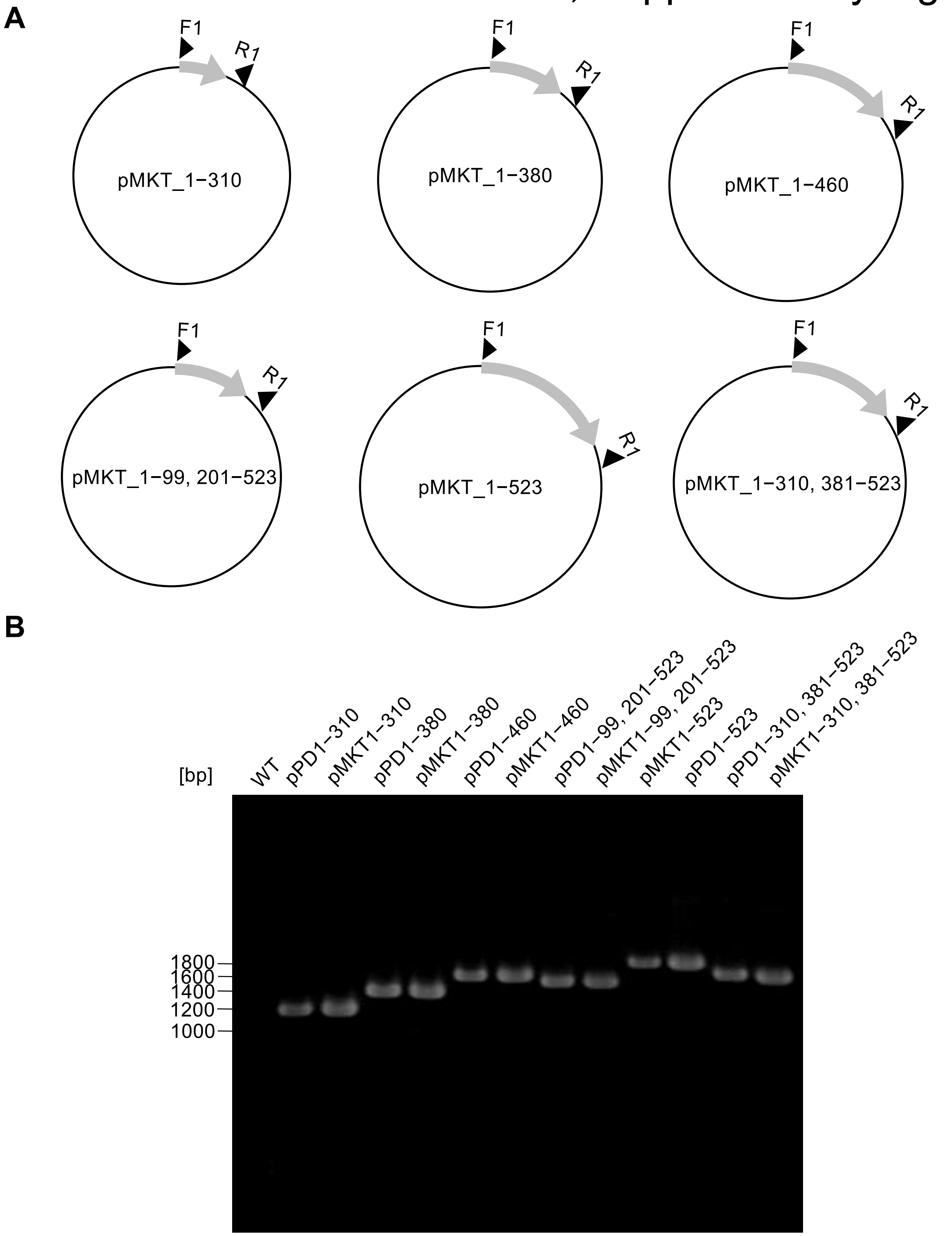

Supplementary Figure 2. The acquisition of CmMYB1 partially truncated strains. **(A)** Schematic representation of the plasmids used for obtaining each CmMYB1 partially truncated strain. Gray arrows indicate *CmMYB1* ORF with different lengths. For example, +1 to +930 bp of *CmMYB1* ORF (+1 indicates the site of start codon) was deleted in pMKT_1−310. **(B)** Confirmation of the transformation. Colonies of *C. merolae* transformants which were used as template DNAs were analyzed by PCR with a set of primers (Table S1), F1/R1. The PCR products were resolved by 0.8% agarose gel electrophoresis. The positions of a molecular size marker are indicated as bp at the left. The size of each PCR product been detected is shown below as predicted. pPD1−310 group: 1133 bp, pPD1−380 group: 1343 bp, pPD1−460 group: 1583 bp, pPD1−99, 201−523 group: 1469 bp, pPD1−523: 1772 bp, pPD1−310, 381−523: 1562 bp. Plasmids used for obtaining transformants were used as positive controls for each group. WT group using WT genomic DNA as a template was used as a negative control.

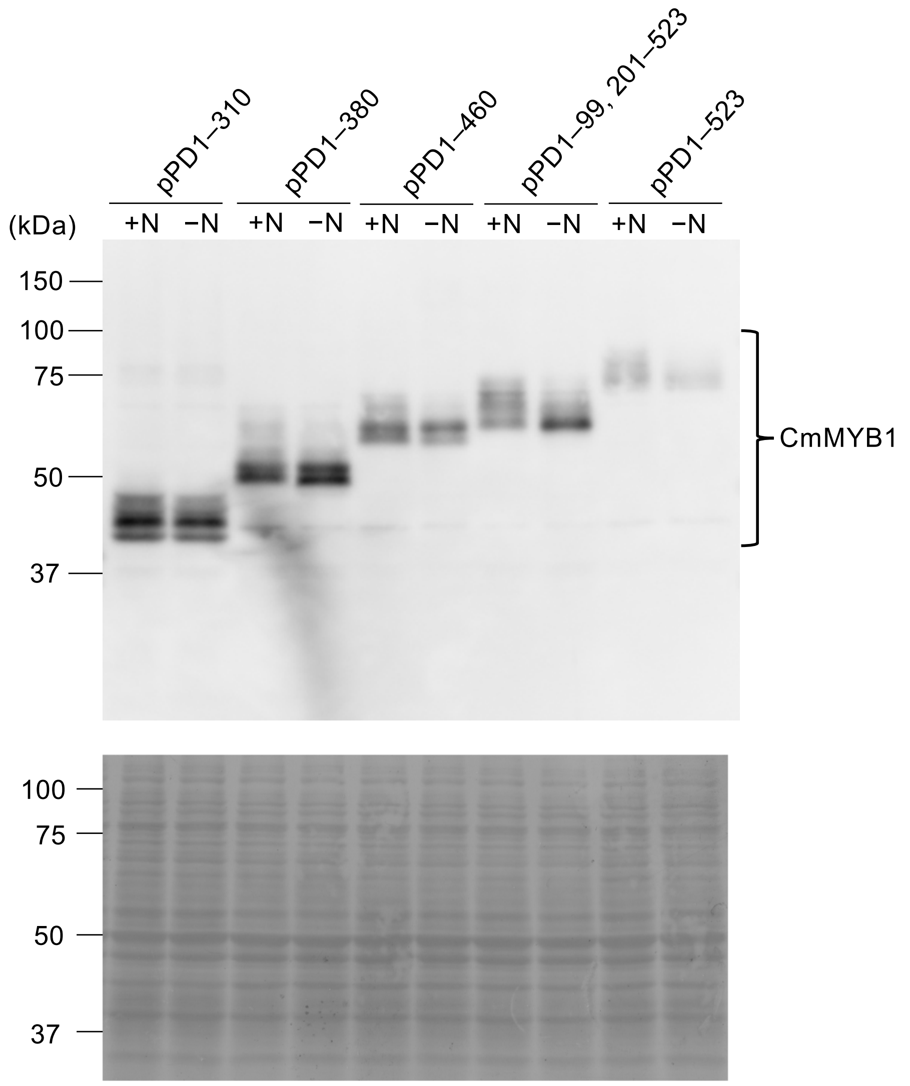

Supplementary Figure 3. Confirmation of the expression of partially truncated CmMYB1s. Aliquots containing 16.6 µg of total proteins from pPD1−310, pPD1−380, pPD1−460, pPD1−99, 201−523, and pPD1−523 cells were separated by 10% SDS-PAGE and analyzed by immunoblot analysis with an anti-FLAG antibody (Wako). Brace indicates the position of FLAG-tagged CmMYB1. The positions of molecular size markers are indicated in kilodaltons (kDa) at the left. The predicted protein sizes were as follows: pPD1−310: 39.1 kDa; pPD1−380: 46.5 kDa; pPD1−460: 55.5 kDa; pPD1−99, 201−523: 50.8 kDa; pPD1−523: 62.2 kDa. Slight migration delay (approximately 4 kDa) was observed in pPD1−380, pPD1−460, and pPD1−99, 201−523. A 12-kDa migration delay was observed in pPD1−523, which was also observed in our previous study (Zhou et al., 2021, doi: 10.2323/JGAM.2020.02.003). Total proteins stained with Coomassie Brilliant Blue (CBB) were shown as a loading control (bottom panel).

Zhou et al., Supplementary Figure 4

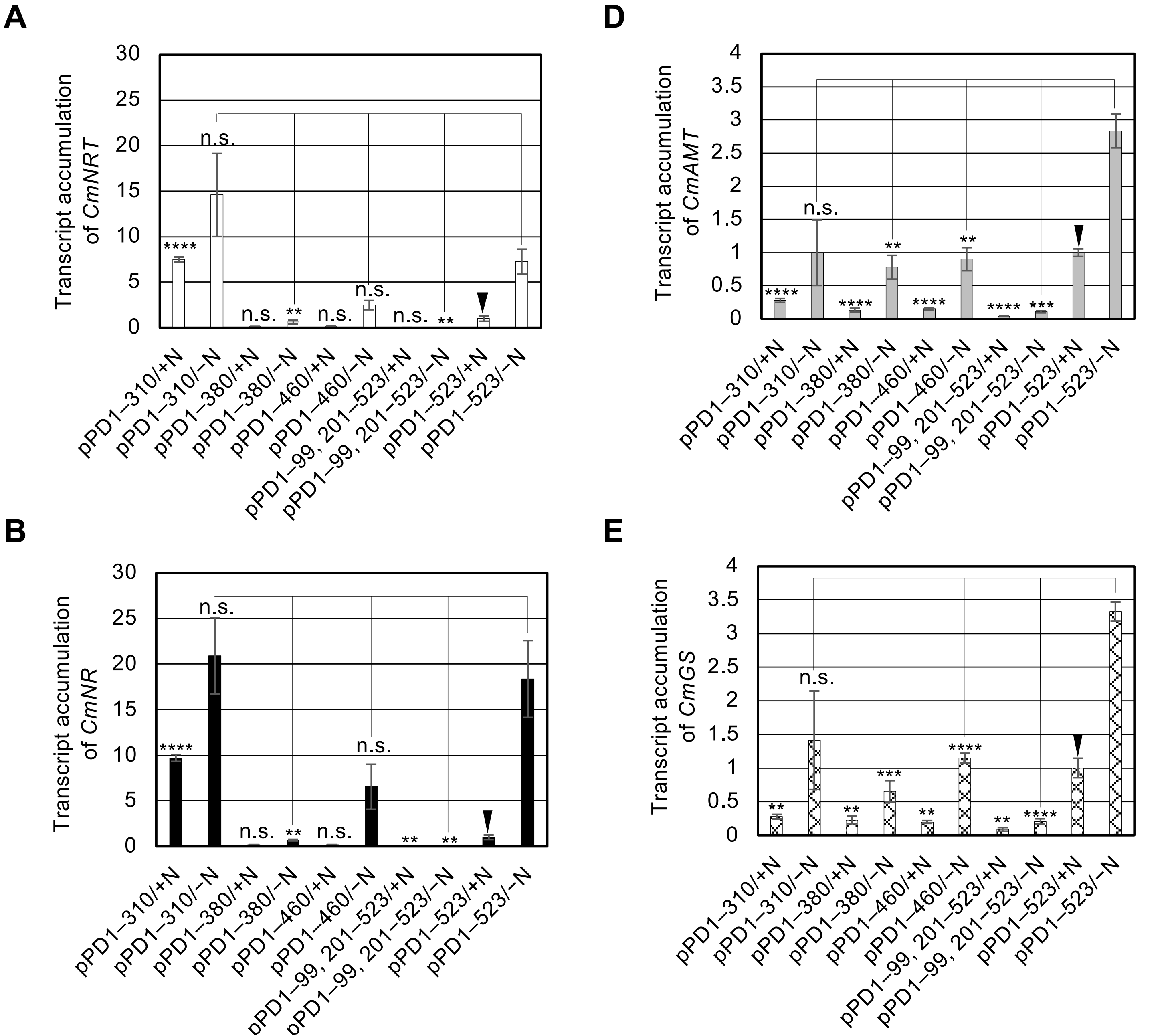

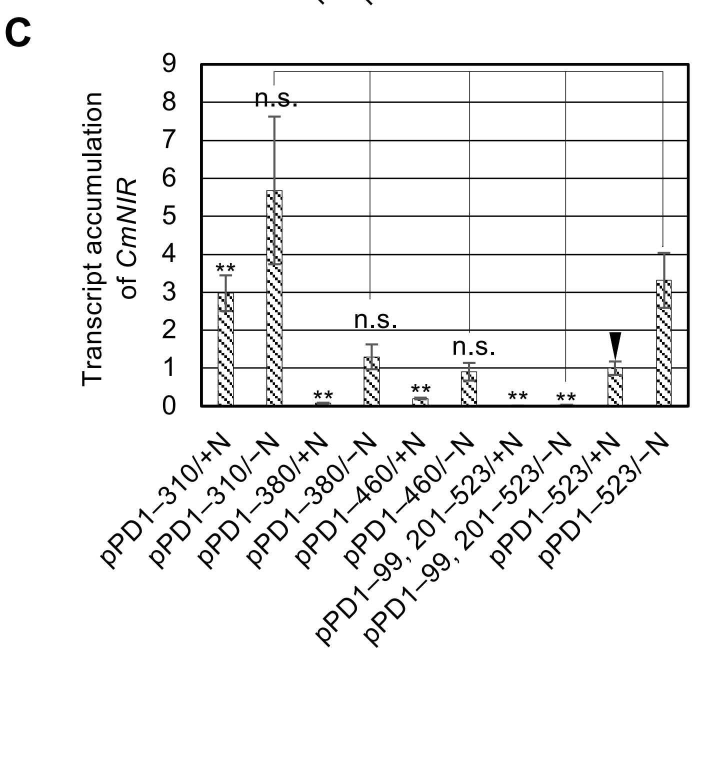

Supplementary Figure 4. Transcript level changes in nitrogen assimilation genes among CmMYB1 partially truncated transformants. **(A)–(E)**, Transcript levels of nitrogen assimilation genes (*CmNRT* in A, *CmNR* in B, *CmNIR* in C, *CmAMT* in D, *CmGS* in E) among CmMYB1 truncated transformant cells after treating +N or −N for 4 hours. Total RNAs were isolated from the cells and 500 ng of total RNAs from each sample were used for genome DNA removal and cDNA synthesis. Results were from three independent experiments, and data represents transcript accumulation of each gene. Error bars indicate the standard deviation (SD). Significant differences were determined using one-way ANOVA (for *CmNRT* under +N condition, p = 0.00000000015; for *CmNRT* under −N condition, p = 0.0037; for *CmNR* under +N condition, p = 0.000000000043; for *CmNR* under −N condition, p = 0.00091; for *CmNIR* under +N condition, p = 0.000013; for *CmNIR* under −N condition, p = 0.011; for *CmAMT* under +N condition, p = 0.0000000096; for *CmAMT* under −N condition, p = 0.00042; for *CmGS* under +N condition, p = 0.000059; for *CmGS* under −N condition, p = 0.00012) followed by post-hoc tests. The asterisks denote the difference between the “pPD1–523/+N” sample vs. other +N condition samples, “pPD1–523/−N” sample vs. other −N condition samples, respectively (****, $p \leq 0.0001$, ***, $p \leq 0.001$, **, $p \leq 0.01$, n.s., $p > 0.0125$).

Zhou et al., Supplementary Figure 5

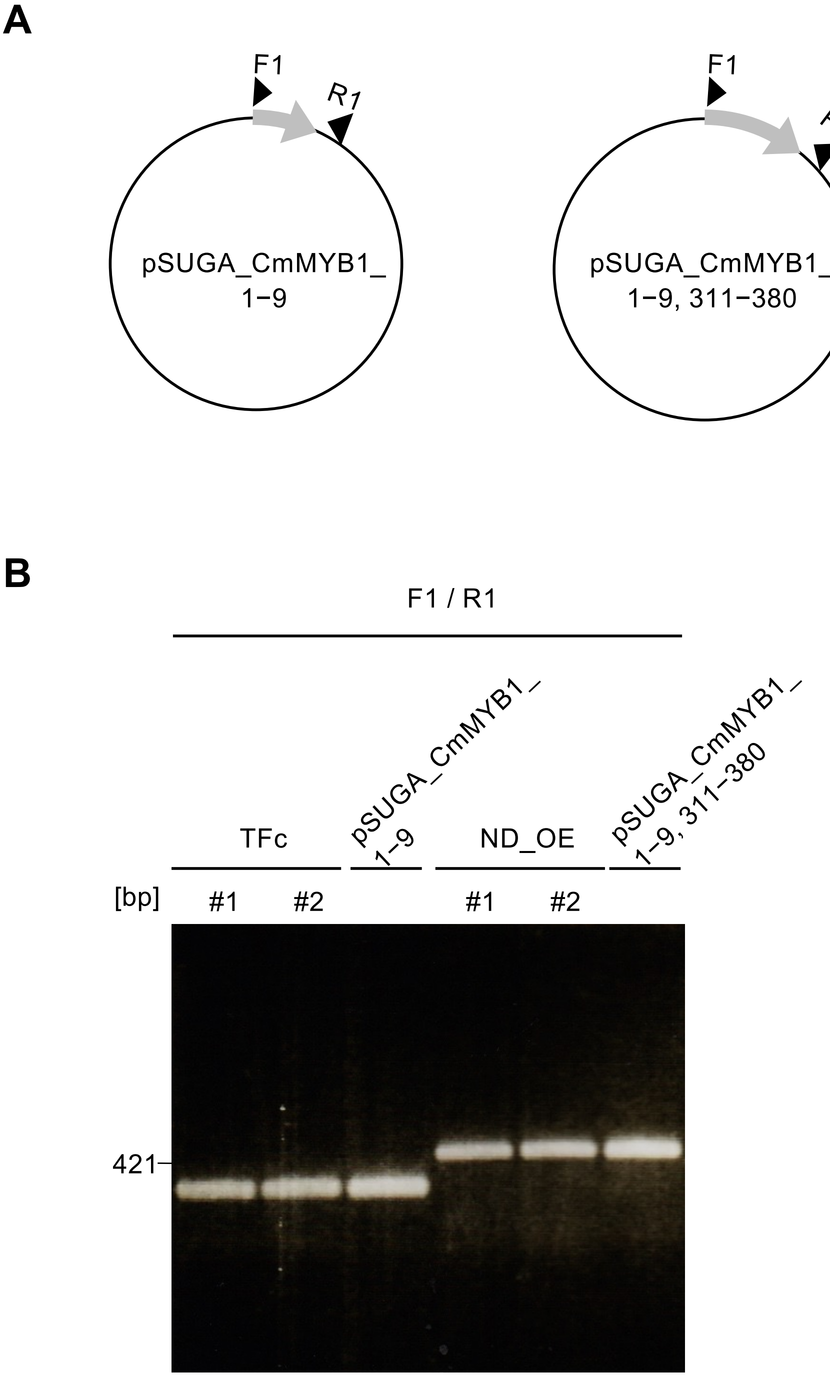

Supplementary Figure 5. Acquisition of ND overexpressing strain (ND_OE) and its control strain (TFc). **(A)** Schematic representation of the plasmids used for obtaining ND_OE and TFc. The plasmids used for obtaining TFc and ND_OE are represented as pSUGA_CmMYB1_1−9 and pSUGA_CmMYB1_1−9, 311−380, respectively. Gray arrows indicate +1 to +27 bp of *CmMYB1* ORF in pSUGA_CmMYB1_1−9 (+1 indicates the site of start codon), +1 to +27 bp, and +931 to +1140 bp of *CmMYB1* ORF in pSUGA_CmMYB1_1−9, 311−380, respectively. **(B)** Confirmation of the transformation. Colonies of *C. merolae* transformants which were used as template DNAs were analyzed by PCR with a set of primers (Table S1), F1/R1. The PCR products were resolved by 0.8% agarose gel electrophoresis. The positions of a molecular size marker are indicated as bp at the left. Size of each PCR product been detected is shown below as predicted. TFc group: 288 bp, ND_OE group: 498 bp. pSUGA_CmMYB1_1−9 and pSUGA_CmMYB1_1−9, 311-380 were used as positive control for TFc and ND_OE, respectively.

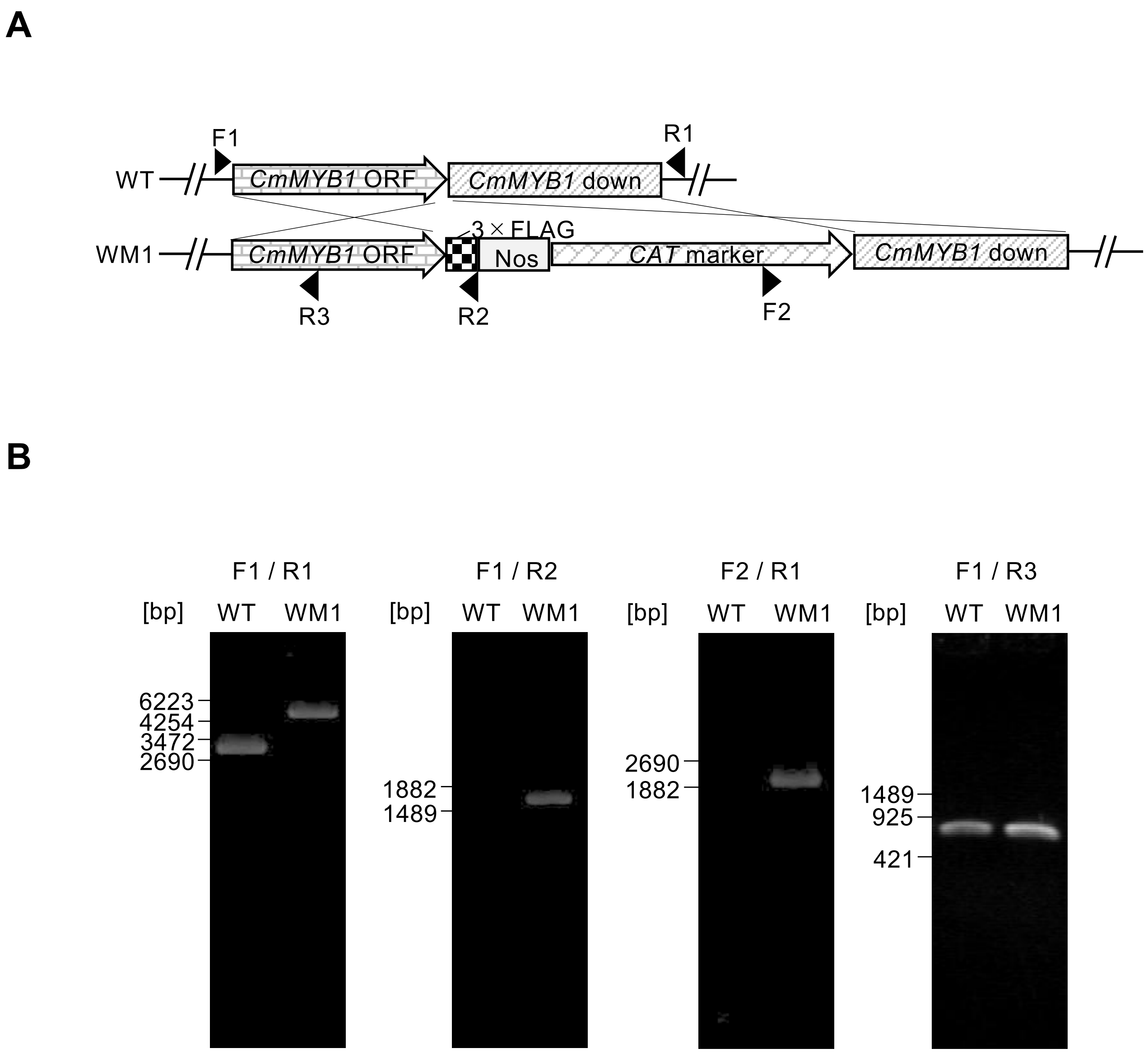

Supplementary Figure 6. Acquisition of FLAG-tagged CmMYB1 strain, WM1. **(A)** Schematic representation of the *CmMYB1* ORF in WT (the parental strain) and WM1 strains. Horizontal and diagonal brick arrows indicate *CmMYB1* and the marker chloramphenicol acetyltransferase, *CAT* gene (*CAT* marker), respectively. Large checkerboard square indicates FLAG epitope tag region. The nopaline synthase (Nos) terminator is represented as "Nos" and *CmMYB1* terminator is represented as "*CmMYB1* down". The positions of primers for PCR are shown with arrowheads. **(B)** Confirmation of the transformation. The genomic DNAs from WT and WM1 were analyzed by PCR with a set of primers (Table S1), F1/R1 (Left), F1/R2 (Second), F2/R1 (Third), or F1/R3 (Right). The PCR products were resolved by 0.8% agarose gel electrophoresis. The positions of a molecular size marker are indicated as bp at the left. Size of each PCR product been detected is shown below as predicted. WT group: 3126 bp, WM1 group: 5217 bp (Left); WT group: no purification, WM1: 1762 bp (the second panel); WT group: no purification, WM1: 2052 bp (the third panel); WT group: 627 bp, WM1 group: 627 bp (Right).

Zhou et al., Supplementary Figure 7

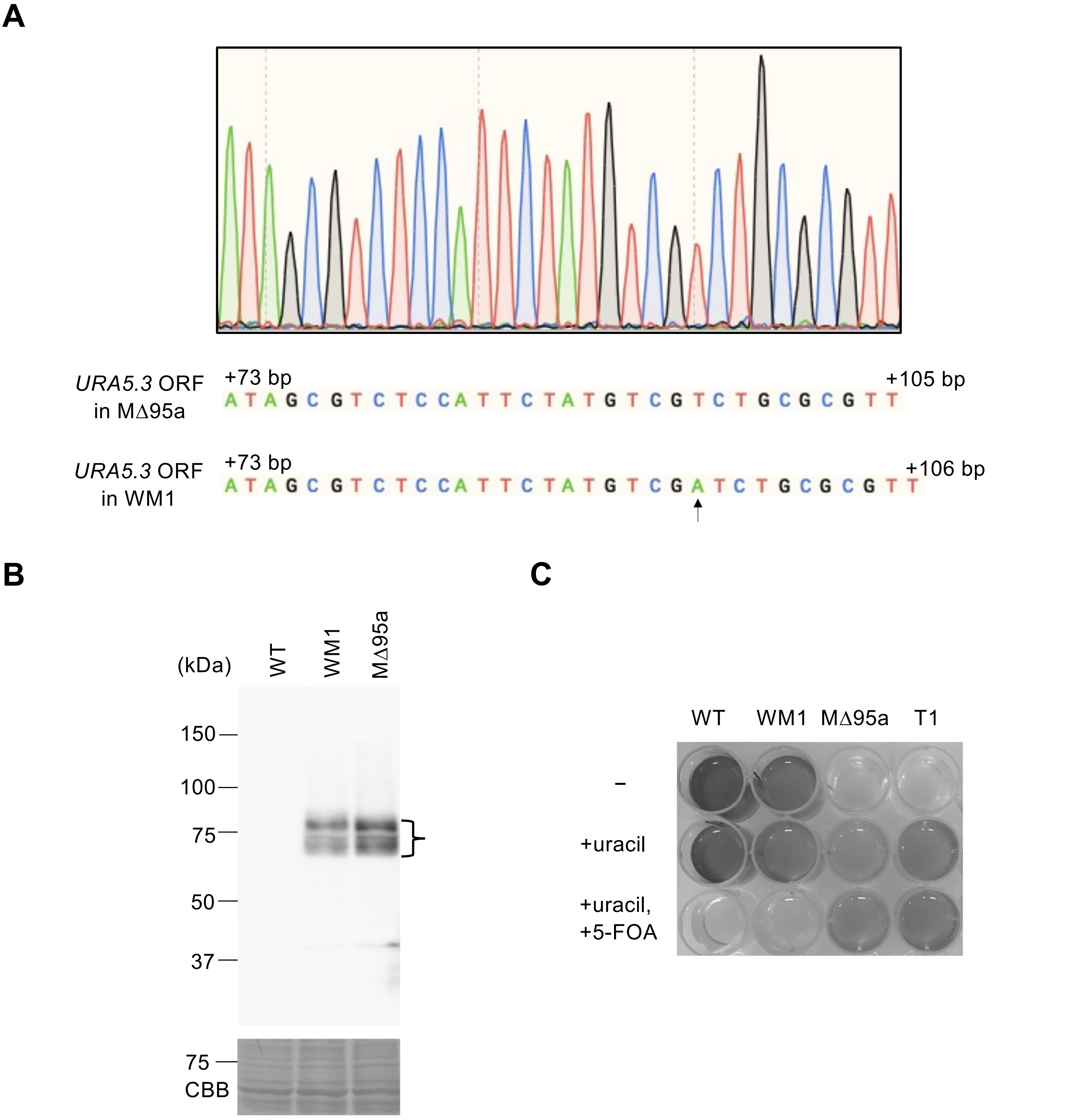

Supplementary Figure 7. Acquisition of uracil auxotrophic strain, MΔ95a. **(A)** Sequence analysis of *URA5.3* ORF in WM1 (parental strain) and MΔ95a. The sequence result shown in the top panel was from MΔ95a. The lost base is pointed by a black arrow. **(B)** Confirmation of FLAG-tagged CmMYB1 expression in WM1 and MΔ95a. Aliquots containing 16.6 μg of total proteins from the WM1 and MΔ95a cells were separated by 10% SDS-PAGE and analyzed by immunoblot analysis with an anti-FLAG antibody (Wako). Protein extracted from WT cells was used as a negative control. Braces indicate the position of FLAG-tagged CmMYB1. The positions of molecular size markers are indicated in kilodaltons (kDa) at the left. FLAG-tagged CmMYB1 was expressed almost equally in WM1 (1) and MΔ95a (1.46) strains. Total proteins (≈75 kDa) stained with CBB were shown as a loading control (bottom panel). **(C)** Uracil auxotrophy of MΔ95a strain. Stationary phase of WM1 and MΔ95a cells were diluted into OD_{750} = 0.1 and incubated for 7 days prior to photography. "–" represents MA2 medium. "+ uracil" represents MA2 medium with 0.5 mg ml^{-1} uracil. "+ uracil, + 5-FOA" represents MA2 medium with 0.5 mg ml^{-1} uracil, 0.8 mg ml^{-1} 5-FOA. MΔ95a could not survive without uracil, whereas WM1 cells could grow in MA2 only medium but died in the medium with 5-FOA, indicating that MΔ95a could be used as a host strain for transformation. WT and T1 cells were treated similarly and used as positive control.

Zhou et al., Supplementary Figure 8

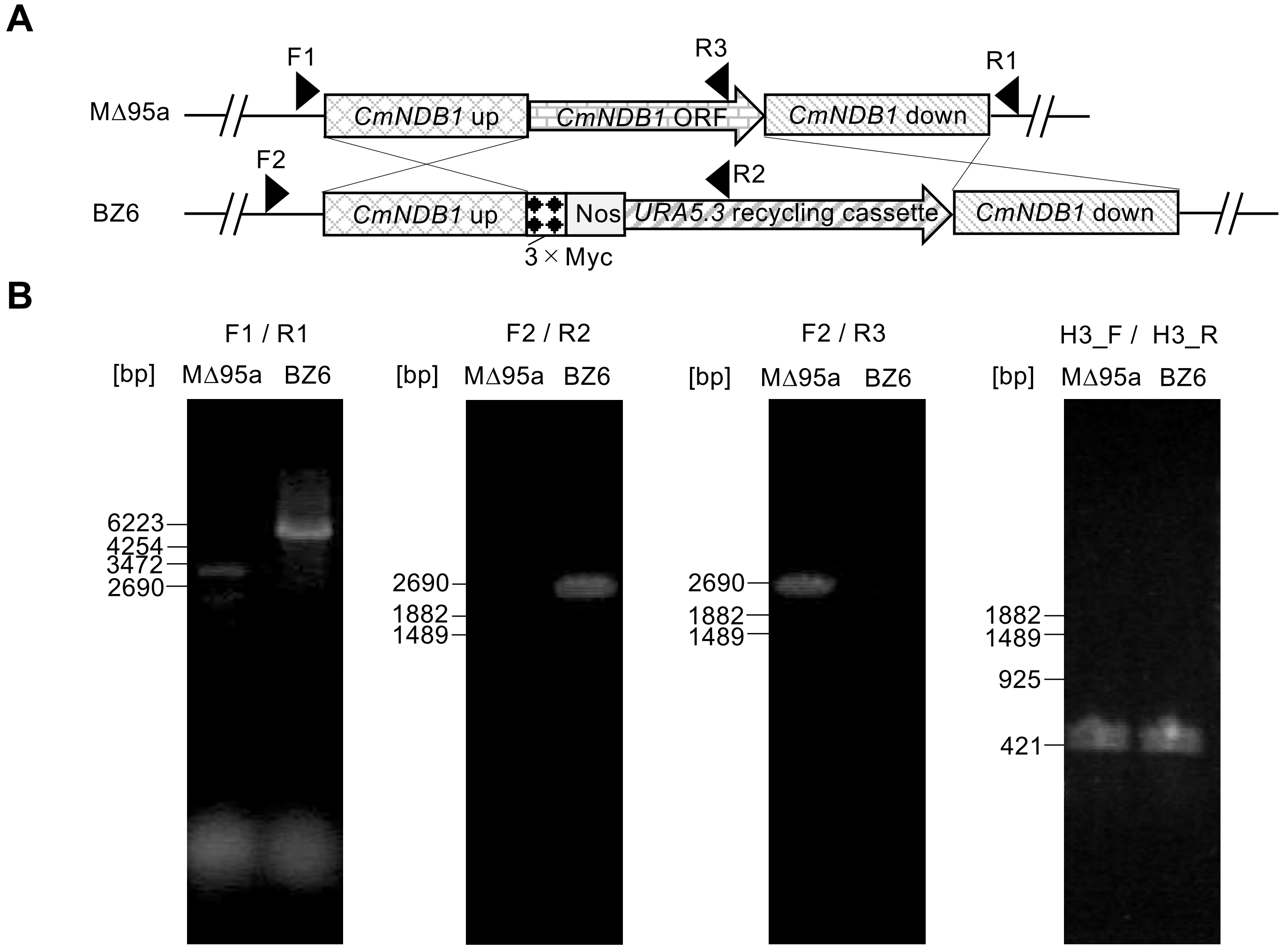

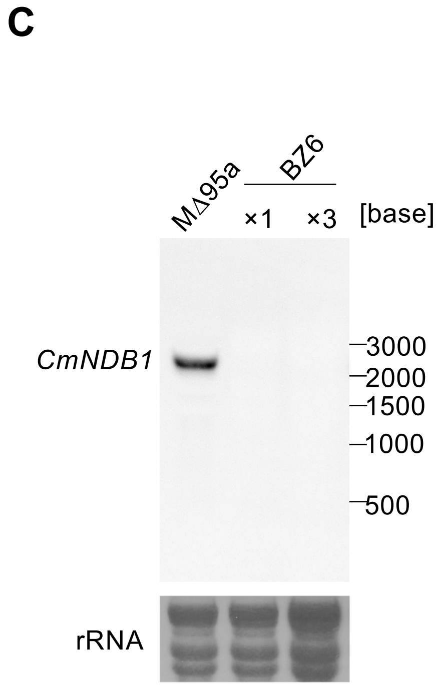

Supplementary Figure 8. Acquisition of *CmNDB1* knockout strain, BZ6. **(A)** Schematic representation of the *CmNDB1* ORF in MΔ95a (the parental strain) and BZ6 strains. Horizontal brick arrow and diagonal stripe arrow indicate *CmNDB1* ORF (*CmNDB1* ORF) and the marker UMP synthase gene (*URA5.3*) contained recycling cassette (*URA5.3* recycling cassette), respectively. Solid diamond grid square indicates Myc epitope tag region. Nos terminator is represented as "Nos" and promoter and terminator of *CmNDB1* are represented as "*CmNDB1* up" and "*CmNDB1* down," respectively. The positions of primers for PCR are shown with arrowheads. **(B)** Confirmation of the transformation. The genomic DNAs from MΔ95a and BZ6 were analyzed by PCR with a set of primers (Table S1), F1/R1 (Left), F2/R2 (Second), F2/R3 (Third), and H3_F/H3_R (Right). The H3 gene is not listed in **A** because it is located at a locus different from *CmNDB1* (*CmNDB1* is located on chromosome 18 and the H3 gene is located on chromosome 14). The PCR products were resolved by 0.8% agarose gel electrophoresis. The positions of a molecular size marker are indicated as bp at the left. The size of each PCR product been detected is shown below as predicted. MΔ95a group: 3070 bp, BZ6 group: 6058 bp (Left); MΔ95a group: no purification, BZ6 group: 2426 bp (the second panel); MΔ95a group: 2601 bp; BZ6 group: no purification (the third panel); MΔ95a group: 408 bp, BZ6 group: 408 bp (Right). **(C)** Confirmation of the transcript level of *CmNDB1* in BZ6. BZ6 and MΔ95a cells were harvested and total RNAs were prepared from the cells. Total RNAs (3 μg) were then subjected to northern blot analysis with specific probe for *CmNDB1*. Total RNAs (9 μg) from BZ6 cells were used as an overloaded sample (×3) in the analysis. The positions of a molecular size marker are indicated as base at the right. The expression of *CmNDB1* at transcription level was not detected in BZ6 cells. rRNA stained with methylene blue was shown as the loading control (bottom panel).

Zhou et al., Supplementary Figure 9

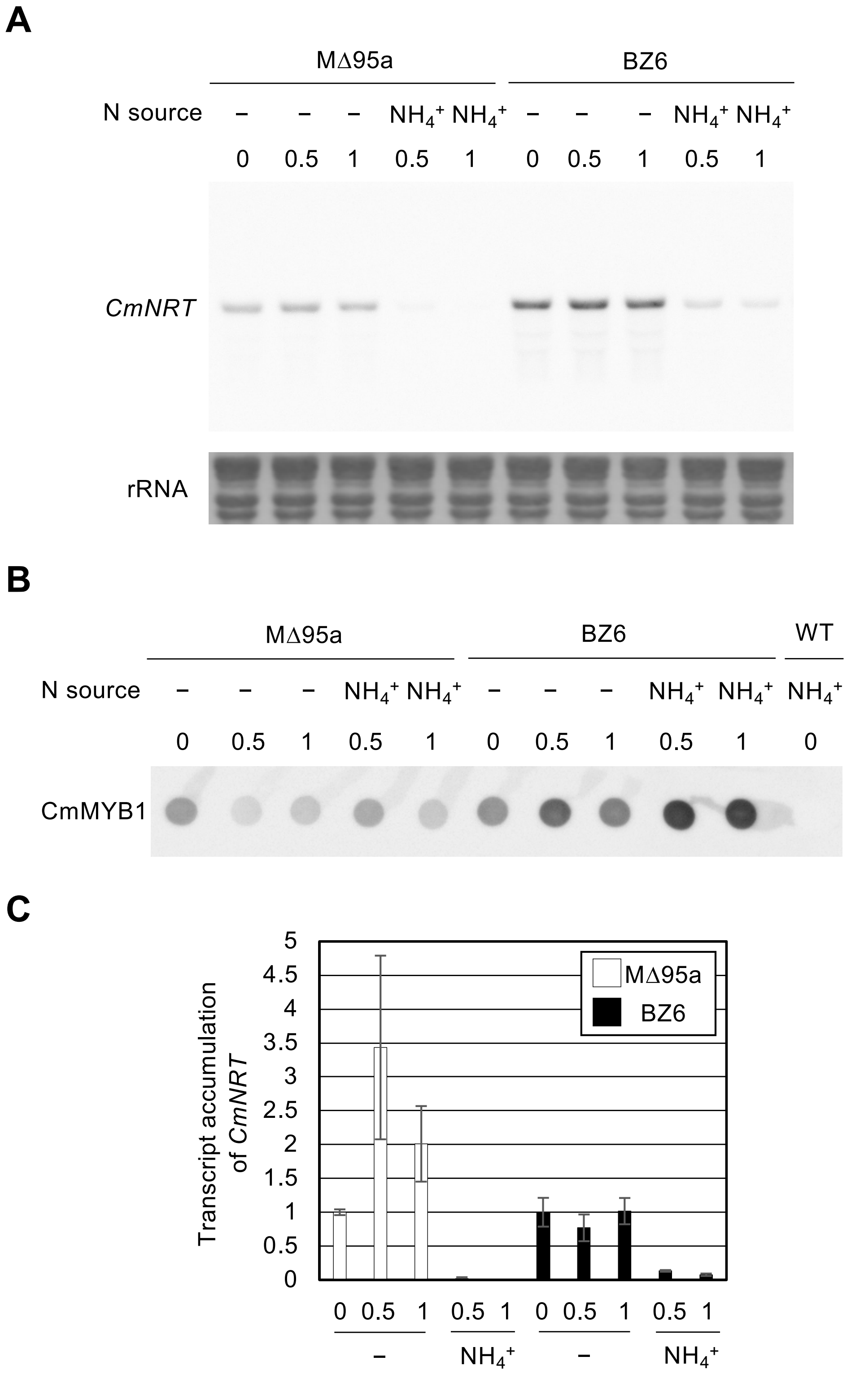

Supplementary Figure 9. Transcript accumulation of *CmNRT* after returning from −N to +N condition in MΔ95a and BZ6. **(A)** Transcript levels of *CmNRT* after returning from −N to +N condition in MΔ95a and BZ6. MΔ95a and BZ6 cells were exposed to −N condition for 4 hours (set time 0), fed ammonium (NH_4^+) at a final concentration of 5 mM or water (−), and sequentially harvested at the indicated time (for instance, "NH_4^+, 0.5" represents cells that were harvested 0.5 hour after feeding NH_4^+). Total RNAs (4.5 µg) were used in northern blot analysis with specific probe for *CmNRT*. The same experiments were performed thrice, independently. **(B)** Protein levels of FLAG-tagged CmMYB1 in MΔ95a and BZ6 after returning from −N to +N condition. Aliquots containing 16.6 µg of total proteins from the *C. merolae* cells, harvested under the same condition as in panel (a), were transferred onto a hydrophilized PVDF membrane and analyzed with an anti-FLAG antibody (Wako). Proteins extracted from WT were used as a negative control. The same experiments were performed thrice, independently. **(C)** Transcript accumulation of *CmNRT* in MΔ95a and BZ6. ImageJ (Schneider et al., 2012, doi: 10.1038/nmeth.2089) was used for determining the signal strength of *CmNRT* and CmMYB1 from panels (a) and (b). Transcript accumulation of *CmNRT* was calculated by dividing the strength of *CmNRT* transcript levels by the strength of CmMYB1 protein levels. Values represent the average transcript accumulation of *CmNRT* in three independent experiments. Time 0 values of MΔ95a and BZ6 were set as 1 and compared with the values of other MΔ95a and BZ6 samples, respectively. Sample information was shown in abscissa. For instance, "−, 0.5" represents cells that were harvested 0.5 hour after feeding water.

Zhou et al., Supplementary Figure 10

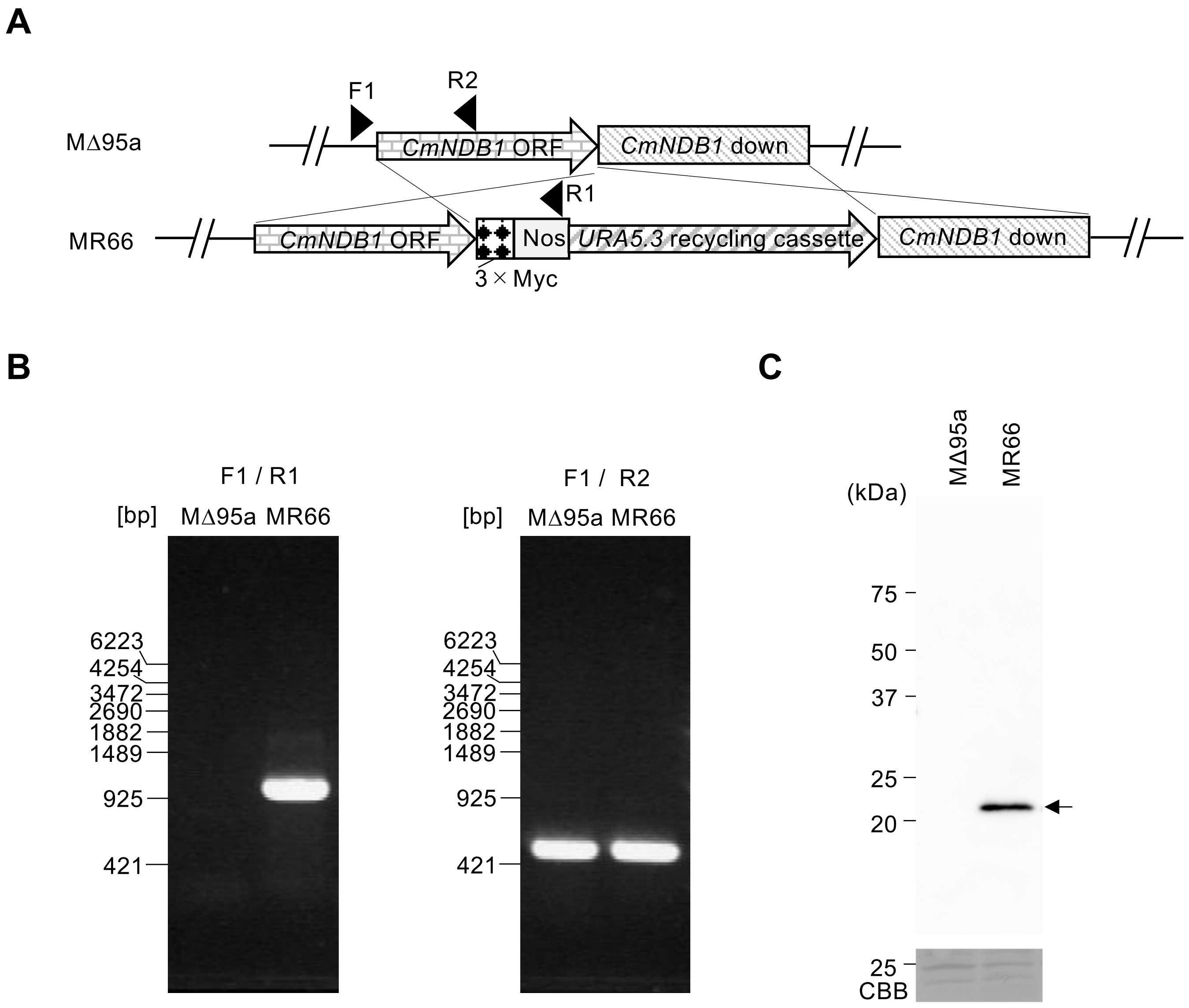

Supplementary Figure 10. The acquisition of Myc-tagged CmNDB1 strain, MR66. **(A)** Schematic representation of the *CmNDB1* ORF in MΔ95a (the parental strain) and MR66 strains. "*CmNDB1* ORF" indicates the 669-bp open reading frame of *CmNDB1*. "*URA5.3* recycling cassette" indicates a 3234-bp fragment used for uracil auxotrophic transformant selection. The solid diamond grid square indicates Myc epitope tag region. Nos terminator is represented as "Nos" and *CmNDB1* terminator is represented as "*CmNDB1* down". The positions of primers for PCR are shown with arrowheads. **(B)** Confirmation of the transformation. The genomic DNAs from MΔ95a and MR66 were analyzed by PCR with a set of primers (Table S1), F1/R1 (Left), F1/R2 (Right). The PCR products were resolved by 0.8% agarose gel electrophoresis. The positions of a molecular size marker are indicated as bp at the left. The size of each PCR product been detected is shown below as predicted. MΔ95a group: no purification, MR66 group: 927 bp (Left); MΔ95a group: 433 bp, MR66 group: 433 bp (Right). **(C)** Confirmation of Myc-tagged CmNDB1 expression in MR66. Aliquots containing 16.6 μg of total proteins from the MΔ95a and MR66 cells were separated by 12% SDS-PAGE and analyzed by immunoblot analysis with an anti-Myc antibody (MBL). Protein extracted from the MΔ95a cells was used as a negative control. The arrow indicates the position of Myc-tagged CmNDB1. The positions of molecular size markers are indicated in kDa at the left. The predicted protein size of Myc-tagged CmNDB1 is 30.2 kDa. Myc-tagged CmNDB1 was expressed only in MR66, but not in MΔ95a with a slight migration shift (approximately 7 kDa) . Total proteins (≈25 kDa) stained with CBB were shown as a loading control (bottom panel).

ND amino acid sequence

+311 +380
SCSEAPQFPETYSSVQVSSSLETKIGEMLPSSYSPVNRCMLTVAHSPLSRCSSSGSLTSLKDGVGKLRVR

ND amino acid sequence

+311 +380
SCSEAPQFPETYSSVQVSSSLETKIGEMLPSSYSPVNRCMLTVAHSPLSRCSSSGSLTSLKDGVGKLRVR

Supplementary Figure 11. Phosphorylation sites of ND. Predicted phosphorylation sites (Serine: S, Threonine: T) of ND are shown in blue in the upper panel. Phosphorylation sites of ND detected by LC-MS/MS are shown in red in the lower panel.
